# The role of host DNA ligases in hepadnavirus covalently closed circular DNA formation

**DOI:** 10.1371/journal.ppat.1006784

**Published:** 2017-12-29

**Authors:** Quanxin Long, Ran Yan, Jieli Hu, Dawei Cai, Bidisha Mitra, Elena S. Kim, Alexander Marchetti, Hu Zhang, Soujuan Wang, Yuanjie Liu, Ailong Huang, Haitao Guo

**Affiliations:** 1 Department of Microbiology and Immunology, Indiana University School of Medicine, Indianapolis, Indiana, United States of America; 2 Institute for Viral Hepatitis, Key Laboratory of Molecular Biology on Infectious Diseases, Ministry of Education, the Second Affiliated Hospital of Chongqing Medical University, Chongqing, China; University of California, San Diego, UNITED STATES

## Abstract

Hepadnavirus covalently closed circular (ccc) DNA is the *bona fide* viral transcription template, which plays a pivotal role in viral infection and persistence. Upon infection, the non-replicative cccDNA is converted from the incoming and *de novo* synthesized viral genomic relaxed circular (rc) DNA, presumably through employment of the host cell’s DNA repair mechanisms in the nucleus. The conversion of rcDNA into cccDNA requires preparation of the extremities at the nick/gap regions of rcDNA for strand ligation. After screening 107 cellular DNA repair genes, we herein report that the cellular DNA ligase (LIG) 1 and 3 play a critical role in cccDNA formation. Ligase inhibitors or functional knock down/out of LIG1/3 significantly reduced cccDNA production in an *in vitro* cccDNA formation assay, and in cccDNA-producing cells without direct effect on viral core DNA replication. In addition, transcomplementation of LIG1/3 in the corresponding knock-out or knock-down cells was able to restore cccDNA formation. Furthermore, LIG4, a component in non-homologous end joining DNA repair apparatus, was found to be responsible for cccDNA formation from the viral double stranded linear (dsl) DNA, but not rcDNA. In conclusion, we demonstrate that hepadnaviruses utilize the whole spectrum of host DNA ligases for cccDNA formation, which sheds light on a coherent molecular pathway of cccDNA biosynthesis, as well as the development of novel antiviral strategies for treatment of hepatitis B.

## Author summary

Hepadnavirus cccDNA is the persistent form of viral genome, and in terms of human hepatitis B virus (HBV), cccDNA is the basis for viral rebound after the cessation of therapy, as well as the elusiveness of a cure with current medications. Therefore, the elucidation of molecular mechanism of cccDNA formation will aid HBV research at both basic and medical levels. In this study, we screened a total of 107 cellular DNA repair genes and identified DNA ligase 1 and 3 as key factors for cccDNA formation from viral relaxed (open) circular DNA. In addition, we found that the cellular DNA ligase 4 is responsible for converting viral double-stranded linear DNA into cccDNA. Our study further confirmed the involvement of host DNA repair machinery in cccDNA formation, and may reveal new antiviral targets for treatment of hepatitis B in future.

## Introduction

Hepadnavirus specifies a group of hepatotropic viruses that carry a single copy of the partially double stranded relaxed circular (rc) viral DNA genome in the enveloped virion particle [1]. Hepadnavirus infects mammalian and avian hosts with strict species-specific tropism, including human hepatitis B virus (HBV) and duck hepatitis B virus (DHBV) [2]. It is estimated that HBV has infected 2 billion people globally, resulting in more than 250 million chronically infected individuals who are under the risk of cirrhosis and hepatocellular carcinoma (HCC) [3, 4].

Upon infection of an hepatocyte, the hepadnaviral rcDNA genome is delivered into the nucleus and converted into an episomal covalently closed circular (ccc) DNA, which exists as a minichromosome and serves as viral mRNA transcription template [5, 6]. One mRNA species, termed pregenomic (pg) RNA, is packaged into the cytoplasmic nucleocapsid, where the viral polymerase reverse transcribes pgRNA into viral minus strand DNA, followed by asymmetric plus strand DNA synthesis to yield the major rcDNA genome or a minor double stranded linear (dsl) DNA form [7]. The mature nucleocapsid either acquires viral envelope proteins for virion egress, or recycles the viral DNA to the nucleus to replenish the cccDNA reservoir [8]. Therefore, cccDNA is an essential component of the hepadnavirus life cycle for establishing a persistent infection, and cccDNA elimination is an undisputed ultimate goal for a cure of hepatitis B [9]. However, the available drugs for treatment of chronic hepatitis B are rarely curative due to their failure to eliminate cccDNA [10]. Therefore there is an urgent unmet need to fully understand HBV cccDNA biology and develop novel effective treatments to directly target cccDNA formation and maintenance [11, 12].

**Fig 1:**
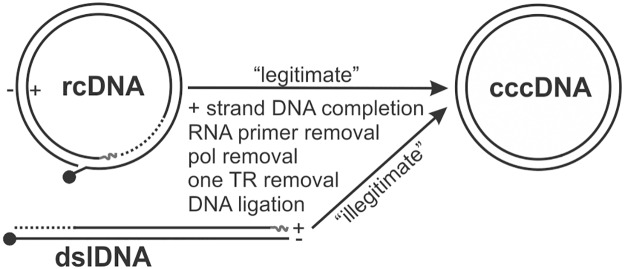
Schematic illustration of the proposed model for hepadnavirus cccDNA formation. Viral polymerase covalently linked to the 5’ end of minus strand (-) DNA of rcDNA and dslDNA is denoted with a filled circle. RNA primer at the 5’ end of plus strand (+) DNA is shown as curved line. The incomplete plus strand DNA is indicated with dashed line. The major obligatory processes in both the legitimate cccDNA formation from rcDNA precursor and the illegitimate cccDNA formation from dslDNA are listed.

Unlike the episomal circular genomes of other DNA viruses, such as papillomaviruses and polyomaviruses [13, 14], HBV cccDNA does not undergo semiconservative replication, but is mainly converted from rcDNA [1]. The molecular mechanism by which rcDNA is converted into cccDNA remains obscure. Comparing the major differences between rcDNA and cccDNA (Fig 1), a series of well-orchestrated biological reactions are required to cope with the terminal molecular peculiarities of rcDNA during cccDNA formation, including: 1) completion of viral plus strand DNA synthesis; 2) removal of the 5’-capped RNA primer at the 5’ terminus of plus strand DNA; 3) removal of viral polymerase covalently attached to the 5’ end of minus strand DNA; 4) removal of one copy of the terminal redundancy on minus strand DNA; 5) ligation of both strands to generate the wildtype cccDNA [5, 9]. Previous studies by us and others have identified a rcDNA species without the viral polymerase attachment on the minus strand DNA, namely deproteinized rcDNA (DP-rcDNA) or protein-free rcDNA (PF-rcDNA), which is a putative functional precursor for cccDNA [15, 16]. The molecular mechanism underlying rcDNA deproteinization is largely unknown. Previous studies have demonstrated that the host tyrosol-DNA phosphodiesterase 2 (TDP2) is able to unlink the covalent bond between viral polymerase and rcDNA *in vitro*, but its role in cccDNA formation remains controversial [17, 18]. We have further demonstrated that DP-rcDNA is produced in cytoplasmic viral capsid and transported into nucleus by cellular karyopherins [19]. Upon nuclear delivery of DP-rcDNA, it is hypothesized that the host functions, most likely the DNA repair machinery, recognize the gaps/nicks in rcDNA as DNA breaks (damages), and repair it into the perfect circular cccDNA [5, 9, 20]. Such an assumption has only been theoretically conceivable and has not been experimentally confirmed until a recent study reported that host DNA polymerase κ (POLK) is involved in HBV cccDNA formation during *de novo* infection [21]. In addition, hepadnaviral dslDNA can also be converted into cccDNA through illegitimate self-circularization after preparing the termini for intramolecular ligation, however, dslDNA-derived cccDNA normally carries insertion-deletion mutations (indels) at the junction region, a typical phenotype of host non-homologous end joining (NHEJ) DNA repair activity [22–24]. In line with this, we have previously demonstrated that an NHEJ component Ku80 is responsible for cccDNA formation from DHBV dslDNA [25], thus further confirming the involvement of DNA repair machinery in cccDNA formation.

In order to systematically identify the host factors involved in cccDNA formation, we screened 107 human DNA repair genes for their effects on HBV cccDNA formation through shRNA knock-down in HepDES19 cells, and selected gene candidates (screen hits) for validation by functional inhibitions in multiple cell systems and assays. We report herein that the cellular DNA ligase (LIG) 1 and 3 are responsible for hepadnavirus cccDNA formation from rcDNA, and the conversion of dslDNA to cccDNA requires LIG4. Such findings will shed light on the molecular mechanism of cccDNA biosynthesis and suggest novel antiviral targets for treatment of chronic hepatitis B.

## Results

### Screen for cellular DNA repair genes involved in cccDNA formation

We screened a total of 107 cellular DNA repair genes for their effects on HBV cccDNA production by shRNA knock-down (see Materials and methods for detailed screening procedures), and the primary screening result is summarized in S1 Fig. The screened genes were grouped based on their primarily associated DNA repair pathways. Knock-down of 8 genes showed more than 50% reduction of cccDNA compared to control knock-down, including LIG1 and LIG3. The low hit rate of the shRNA screen may be due to the incomplete depletion of the targeted genes and/or functional redundancy of the cellular DNA repair genes/pathways. Surprisingly, a large number of gene knock-down resulted in cccDNA upregulation. Such unanticipated observation indicates that certain DNA repair genes may negatively regulate cccDNA formation through rcDNA sequestration or even degradation, which awaits further investigations.

We prioritized DNA ligases for functional validation in cccDNA formation for two reasons. Firstly, although the redundant activities of host DNA repair factors may be involved in cccDNA formation, it is likely that the DNA ligation is an essential and final step to seal the nicks/gaps on rcDNA. Secondly, there are only three known DNA ligases in mammalian cells, including LIG1, LIG3, and LIG4 [26], and we have previously ruled out the involvement of NHEJ repair apparatus, which contains LIG4, in rcDNA to cccDNA conversion [25].

### DNA ligase inhibitors block cccDNA formation in nuclear extract

**Fig 2:**
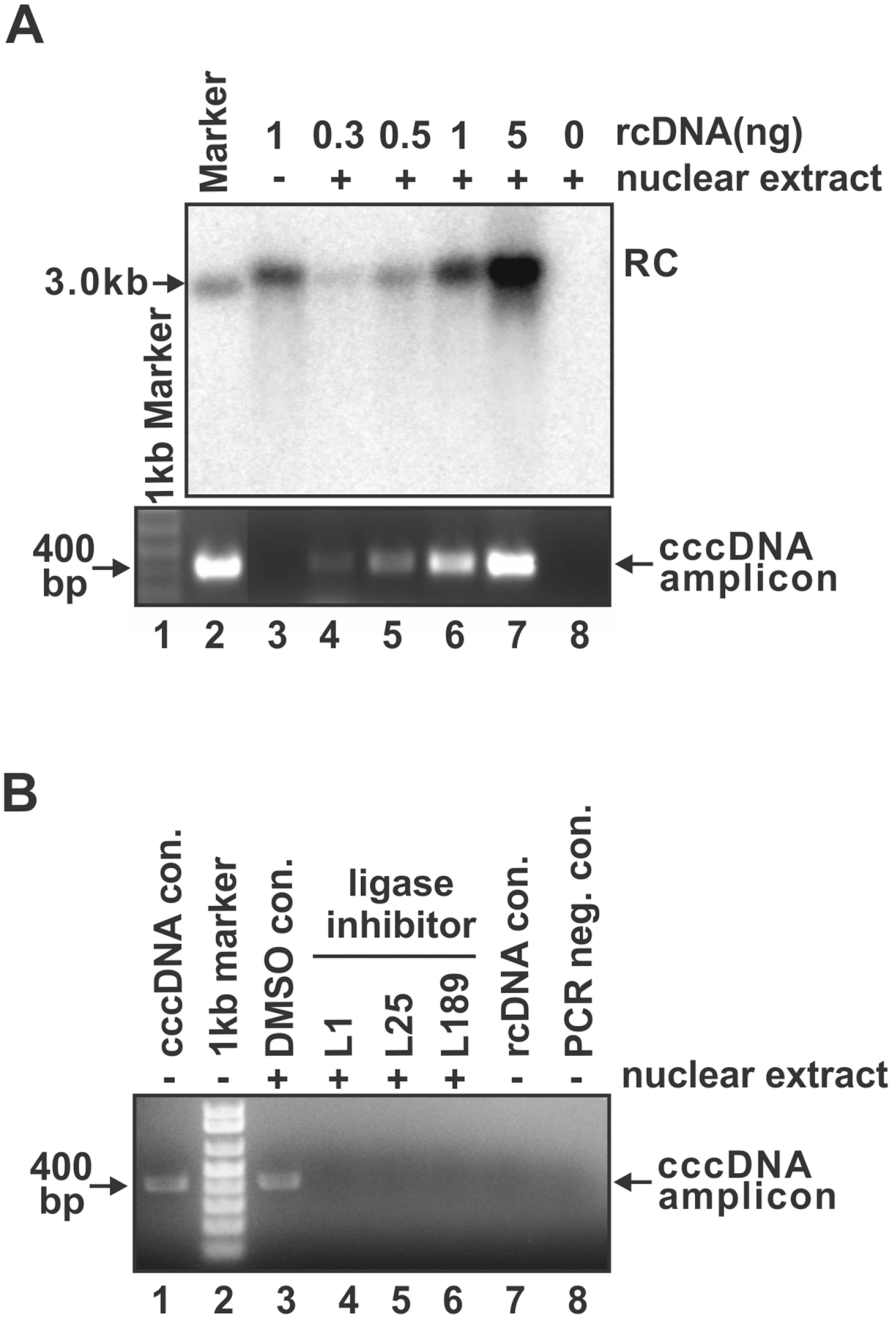
Ligase inhibitor treatment blocked cccDNA formation in nuclear extract. (A) Demonstration of the *in vitro* cccDNA formation assay. The indicated amount of purified DHBV rcDNA were incubated with HepG2 nuclear extract as described in Materials and Methods. After the *in vitro* cccDNA formation reaction, total DNA were extracted and subjected to DHBV Southern blot analysis (top panel) and cccDNA-specific PCR assay (bottom panel). The EcoRI-linearized DHBV unit-length DNA was used as marker in Southern blot and as PCR positive template in cccDNA PCR assay (lane 2). 1 ng of rcDNA alone (lane 3) and nuclear extract alone (lane 8) served as PCR negative controls. (B) The *in vitro* DHBV cccDNA formation reaction was treated with DNA ligases inhibitor L1 (20 μM), L25 (20 μM), or L189 (50 μM), or DMSO solvent, and cccDNA was detected by PCR (lanes 3–6). DHBV cccDNA purified from Dstet5 cells was used as PCR positive control (lane 1). 1 ng of rcDNA without incubation with nuclear extract (lane 7) or without PCR reaction (lane 8) served as negative controls.

In addition to the above cell-based screening assay, considering that the conversion of rcDNA into cccDNA occurs in the cell nucleus where the DNA repair machinery functions, we established an *in vitro* cell-free cccDNA formation system with nuclear protein extract. With this assay, we have tested the ability of cccDNA formation from the purified DHBV virion rcDNA. As shown in Fig 2A, although cccDNA could not be directly detected by Southern blot, we were able to detect cccDNA signal by a sensitive PCR assay with primers targeting the sequences outside of the gap region in DHBV rcDNA (S2A Fig), a similar principle has been used for the quantitative detection of HBV cccDNA by real-time PCR [27]. The assay specificity is sufficient enough to distinguish 0.3 pg of cccDNA from 30 pg of input rcDNA (S2B and S2C Fig). Sequence analysis of the cccDNA amplicons demonstrated a perfect repair of rcDNA gaps in nuclear extract. It is worth noting that the observed cccDNA PCR signal from the above *in vitro* cccDNA formation assay might be derived from rcDNA-like template with one strand being repaired into a closed circular DNA. In line with this, a recent study has identified a HBV rcDNA species with a covalently closed minus strand but an open plus strand through digesting the Hirt DNA samples from cell cultures with 3’→5’ exonuclease I and III (ExoI/III), which is possibly an intermediate during rcDNA to cccDNA conversion [28]. We thus attempted to search for such rcDNA-like species that might be generated during the *in vitro* cccDNA formation reaction by the similar approach. However, neither the minus-strand nor the plus-strand closed circular ssDNA was detected by Southern blot (S3 Fig), indicating that the rcDNA species with covalently closed minus or plus strand in this *in vitro* cccDNA formation assay, if any, is below the detection limit of Southern blot. Further optimization is needed to improve the assay robustness. Nonetheless, the current *in vitro* cell-free cccDNA formation assay may still be able to partly recapitulate the nuclear events during rcDNA to cccDNA conversion, which provides a simple and convenient system to assist the study of DNA repair functions in hepadnavirus cccDNA formation.

To assess the role of host DNA ligases in hepadnavirus cccDNA formation, LIG1/3 inhibitor L1 and L25, and pan ligase inhibitor L189 that inhibit the interaction between human DNA ligases and nicked DNA [29], were added into the *in vitro* cccDNA formation reaction. As shown in Fig 2B, all the ligase inhibitors tested completely blocked the rcDNA-to-cccDNA conversion in nuclear extract.

### Ligase inhibitor L189 inhibited DHBV cccDNA formation in cell cultures

**Fig 3:**
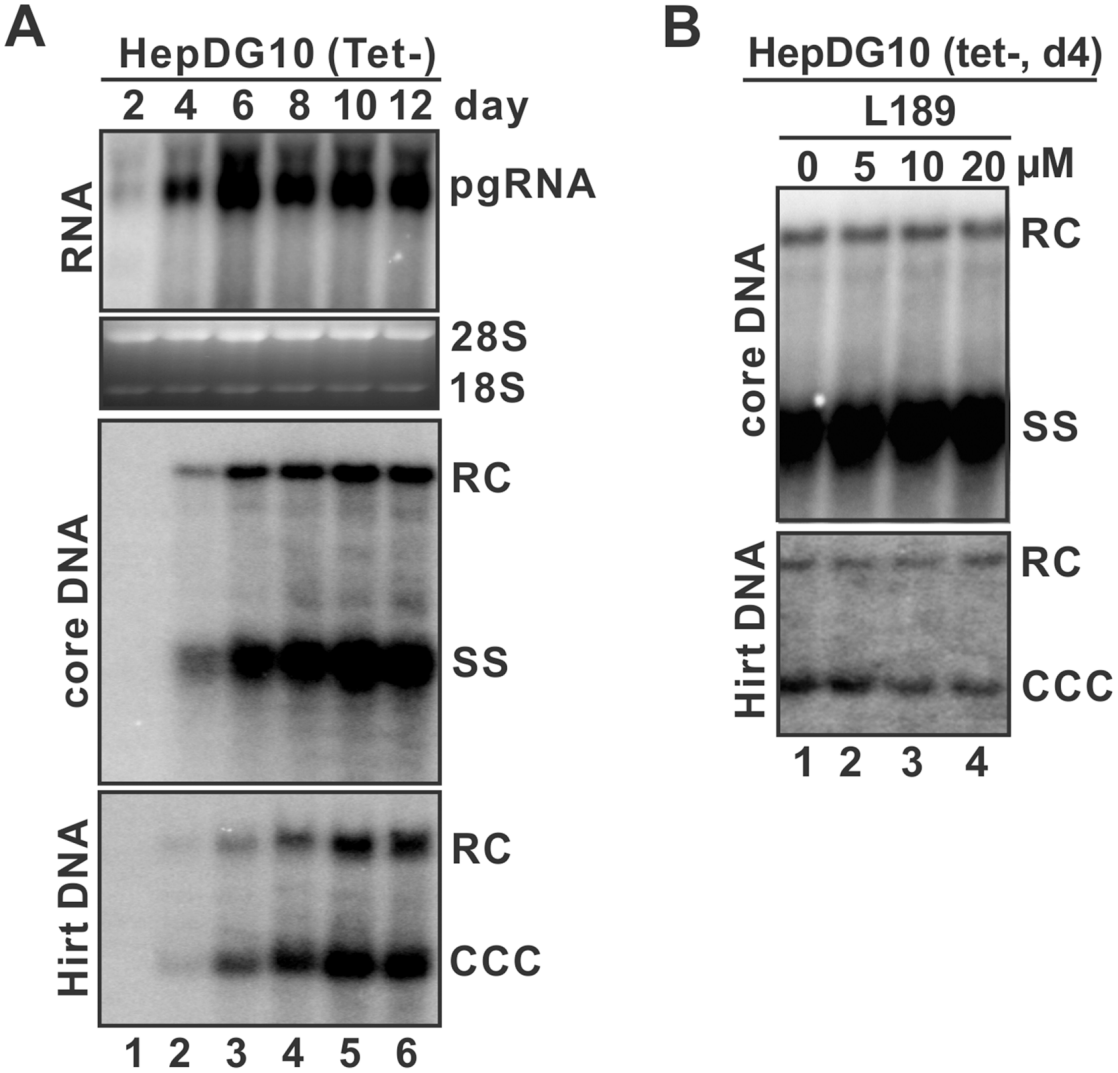
Ligase inhibitor treatment reduced cccDNA formation in HepDG10 cells. (A) Time course kinetics of DHBV pgRNA transcription, core DNA replication, and cccDNA accumulation in the HepG2-based, tet-inducible HepDG10 cells. HepDG10 cells were cultured in tet-free medium and harvested at the indicated post induction time points. DHBV pgRNA was detected by Northern blot. The cytoplasmic DHBV core DNA and whole cell Hirt DNA were analyzed by Southern blot. (B) HepDG10 cells cultured in tet-free medium were left untreated or treated with indicated concentration of L189 for 4 days, followed by Southern blot analyses of viral core DNA and Hirt DNA.

Taking advantage of the fast kinetics and high productivity of DHBV cccDNA formation, we established a tetracycline (tet)-inducible DHBV stable cell line in the background of HepG2 cells, namely HepDG10, to study hepadnavirus cccDNA formation in human cells. As shown in Fig 3A, DHBV pgRNA, core DNA and cccDNA were rapidly and robustly produced in HepDG10 cells upon withdrawal of tet, cccDNA could be detected by Southern blot as early as day 4 post induction and it accumulated to more significant levels after longer induction. The authenticity of the cccDNA band shown on Southern blot was confirmed by heat denaturation and EcoRI linearization (S4 Fig). Considering that HBV cccDNA formation in cell cultures is extremely time-consuming [15], the HepDG10 cell line thus provides a robust and convenient cell-based system for assessing human gene functions in hepadnavirus cccDNA formation through loss-of-function approaches (e.g. chemical inhibitors, gene knock down/out), which the relative short assay window may avoid or reduce the potential cytotoxic effects and/or functional redundancy. Consistent with the results from the *in vitro* nuclear extract-based cccDNA formation assay (Fig 2B), pan-ligase inhibitor L189 also inhibited DHBV cccDNA accumulation in HepDG10 cells without affecting core DNA replication (Fig 3B), further indicating a role of ligase(s) in cccDNA formation. The incomplete inhibition of cccDNA formation by L189 might be due to partial inhibition of cellular ligases under the nontoxic concentrations tested in HepDG10 cells.

### Knock-out of LIG1 or LIG3 inhibited DHBV cccDNA formation

**Fig 4:**
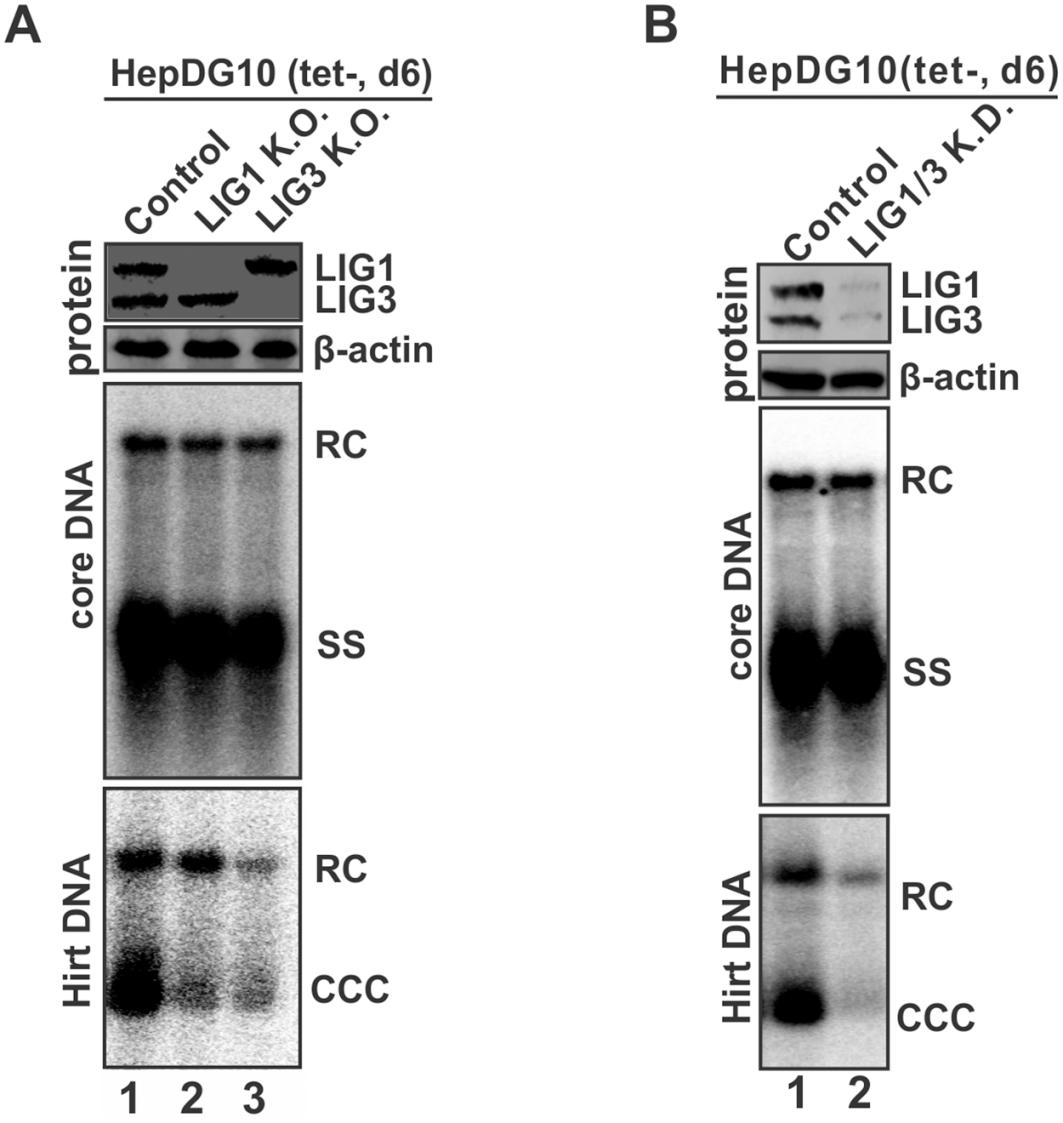
Knock-out of LIG1 and LIG3 reduced DHBV cccDNA in HepDG10 cells. HepDG10 control and ligase single knock-out (K.O.) cells (A) and double knock-down cells (B) were cultured in tet-free medium for 6 days. The levels of LIG1 and LIG3 in each cell lines were determined by Western blot with β-actin serving as loading control. DHBV core DNA and cccDNA were analyzed by Southern blot. The loading amount of Hirt DNA was normalized based on the relative levels of RC DNA (% of control) on core DNA Southern blot. The data presented here are representative of two independent experiments.

To further assess the role of DNA ligases in cccDNA formation, the expression of LIG1 and LIG3 in HepDG10 cells was completely blocked through gene knock-out by CRISPR/Cas9 (Fig 4A, top panel). The indel mutation sequencing result confirmed the disruption of LIG1 and LIG3 gene at genomic DNA level in the established HepDG10-LIG1 K.O. and HepDG10-LIG3 K.O. cells, respectively (S5A, S5B, S6A and S6B Figs). Depletion of LIG1 or LIG3 did not affect the rcDNA production prominently but resulted in a significant reduction of cccDNA in HepDG10 cells (Fig 4A), suggesting that both LIG1 and LIG3 are involved in cccDNA formation. Similar result was observed with another clone of HepDG10-LIG1 K.O. and HepDG10-LIG3 K.O. cells. The incomplete inhibition of cccDNA formation by knocking out either LIG1 or LIG3 might be due to a redundant function between these two DNA ligases. Consistent with this, Sanger sequencing of cccDNA from the control and LIG1/3 knock-out cells showed wild type DHBV sequence between DR1 and DR2.

We have also attempted to CRISPR-out LIG1 and LIG3 simultaneously in HepDG10 cells, but were unable to obtain clones with complete knock-out of both genes, which might be due to that at least one ligase gene is required for cell viability. However, in a single colony-derived cell line with LIG1/3 double knock-down, which the knock-down efficiency was confirmed by both Western blot (Fig 4B, top panel) and T7E1 indel assay (S7 Fig), DHBV cccDNA formation was more profoundly inhibited without affecting core DNA replication (Fig 4B), further confirming the requirement of LIG1/3 in cccDNA synthesis. In addition, the similar phenomena was observed in HepDG10 cells with LIG1/3 single or double knock-down by lentiviral shRNA (S8 Fig).

It is of note that the protein-free rcDNA on Hirt DNA Southern blot was also reduced under LIG1 or LIG3 knock-out (Fig 4, bottom panels). We reason that the concurrent decrease of protein-free rcDNA might be a consequence of cccDNA reduction because the Hirt DNA sample from HepDG10 cells contained a large quantity of nicked cccDNA, which has the indistinguishable electrophoretic mobility as true rcDNA but cannot be mild heat denatured into dslDNA form (S9 Fig). The presence of nicked cccDNA in Hirt extraction has been described in previous studies [16, 30].

### LIG1 and LIG3 are involved in HBV cccDNA formation

**Fig 5:**
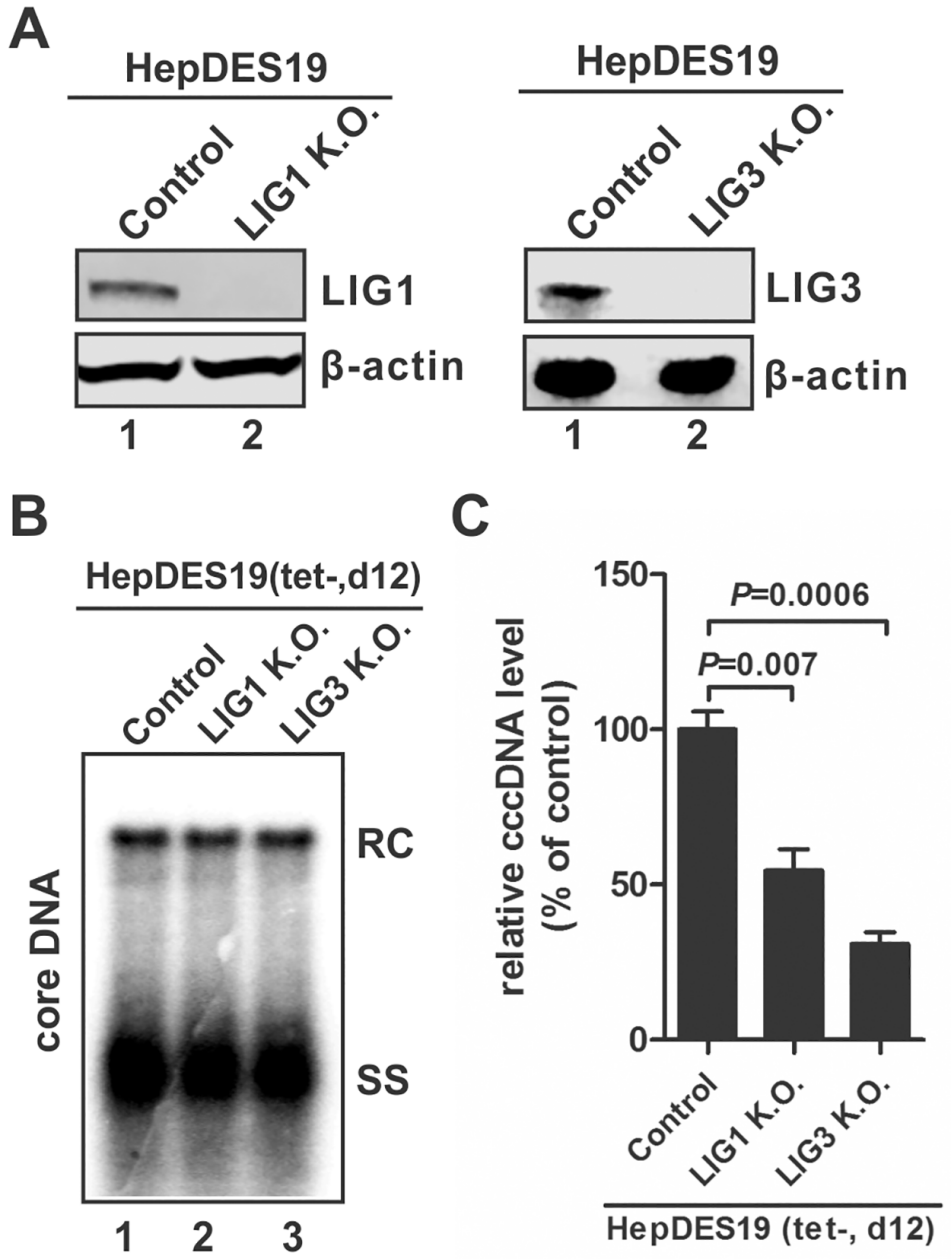
Knock-out of LIG1 or LIG3 reduced HBV cccDNA production in HBV stable cell line. (A) Confirmation of LIG1 and LIG3 knock-out in HepDES19-based LIG1 K.O. cells and LIG3 K.O. cells by Western blot, respectively. (B) HepDES19-based control K.O. cells, LIG1 K.O. cells, and LIG3 K.O. cells were cultured in tet-free medium for 12 days, then the intracellular HBV core DNA was analyzed by Southern blot. (C) HBV Hirt DNA samples extracted from the above cell cultures were heat denatured and digested by PSAD to remove rcDNA, then cccDNA was quantified by qPCR, normalized by mitochondrial DNA and plotted as relative levels (% of control) (mean±SD, n = 3).

To validate the role of LIG1/3 in HBV cccDNA formation, the individual ligase was knocked out in HBV stable cell line HepDES19 cells by CRISPR/Cas9 (Figs 5A, S5A–S5C and S6A–S6C). Upon tet induction, the cytoplasmic rcDNA remained unchanged in LIG1 or LIG3 knock-out cells compared to control knock-out cells (Fig 5B). cccDNA qPCR assay demonstrated a significant reduction of cccDNA in LIG1/3 knock-out HepDES19 cells (Fig 5C). In order to avoid the contamination of rcDNA in cccDNA quantitation, we developed a method to eliminate rcDNA in Hirt DNA samples before qPCR, which involves a 85°C heat denaturation step that selectively denatures rcDNA into single stranded (ss) DNA, followed by Plasmid-safe ATP-dependent DNase (PSAD) treatment to remove non-cccDNA templates (Materials and methods) (S10 Fig).

**Fig 6:**
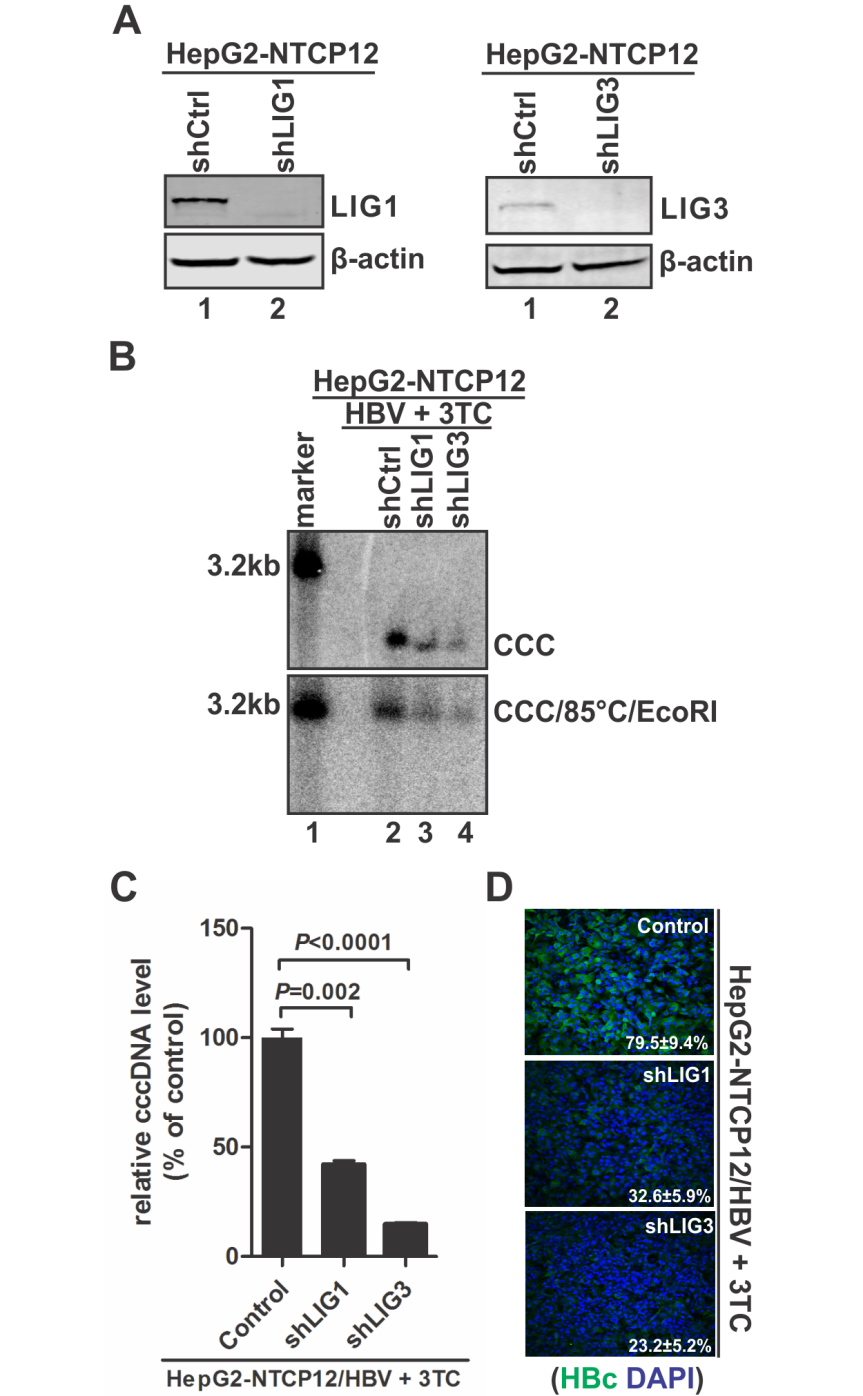
Knock-down of LIG1 or LIG3 reduced HBV cccDNA production in HBV *in vitro* infection system. (A) The knock-down levels of LIG1 and LIG3 in lentiviral shRNA transduced HepG2-NTCP12 cells were assessed by Western blot. (B) The indicated control and LIG1/3 knock-down HepG2-NTCP12 cells were infected by HBV in the presence of 10 μM of 3TC for 8 days. The extracted intracellular HBV cccDNA were directly analyzed by Southern blot (top panel), or heat denatured at 85°C for 5 min, followed by EcoRI linearization and Southern blot analysis (bottom panel). (C) In addition, cccDNA was quantitated by qPCR, normalized by mitochondrial DNA and plotted as relative levels (% of control) (mean±SD, n = 3). (D) The intracellular core protein (HBc) expression was detected by immunofluorescence microscopy. Cell nuclei were counterstained with DAPI. Each image is a representative of five different microscopic fields, the percentage of HBc-positive cells was indicated (Mean ± SD).

During natural infection, hepadnavirus cccDNA is formed from both the invading rcDNA in virion and the newly synthesized rcDNA [1]. Because the DHBV or HBV stable cell line used in above studies only makes cccDNA through the rcDNA recycling pathway, we, thus, further assessed the role of LIG1/3 in HBV infection system. To do so, LIG1/3 expression was stably suppressed in HepG2-NTCP12 cells by lentiviral shRNA (Fig 6A). The control and LIG1/3 knock-down HepG2-NTCP12 cells were infected with HBV particles in the presence of nucleoside analogue 3TC which is known to block *de novo* HBV DNA replication but not the initial cccDNA formation [21, 31], making the system suitable for studying the first round cccDNA formation from the input viruses. Upon infection, the levels of HBV cccDNA and core protein were markedly lower in HepG2-NTCP cells with LIG1 or LIG3 knock-down compared to the control knock-down cells (Fig 6B, 6C and 6D), suggesting that LIG1 and LIG3 are also required for the first round HBV cccDNA formation during *de novo* infection.

### Ectopic expression of LIG1 and LIG3 in knock-out or knock-down cells rescued cccDNA formation

**Fig 7:**
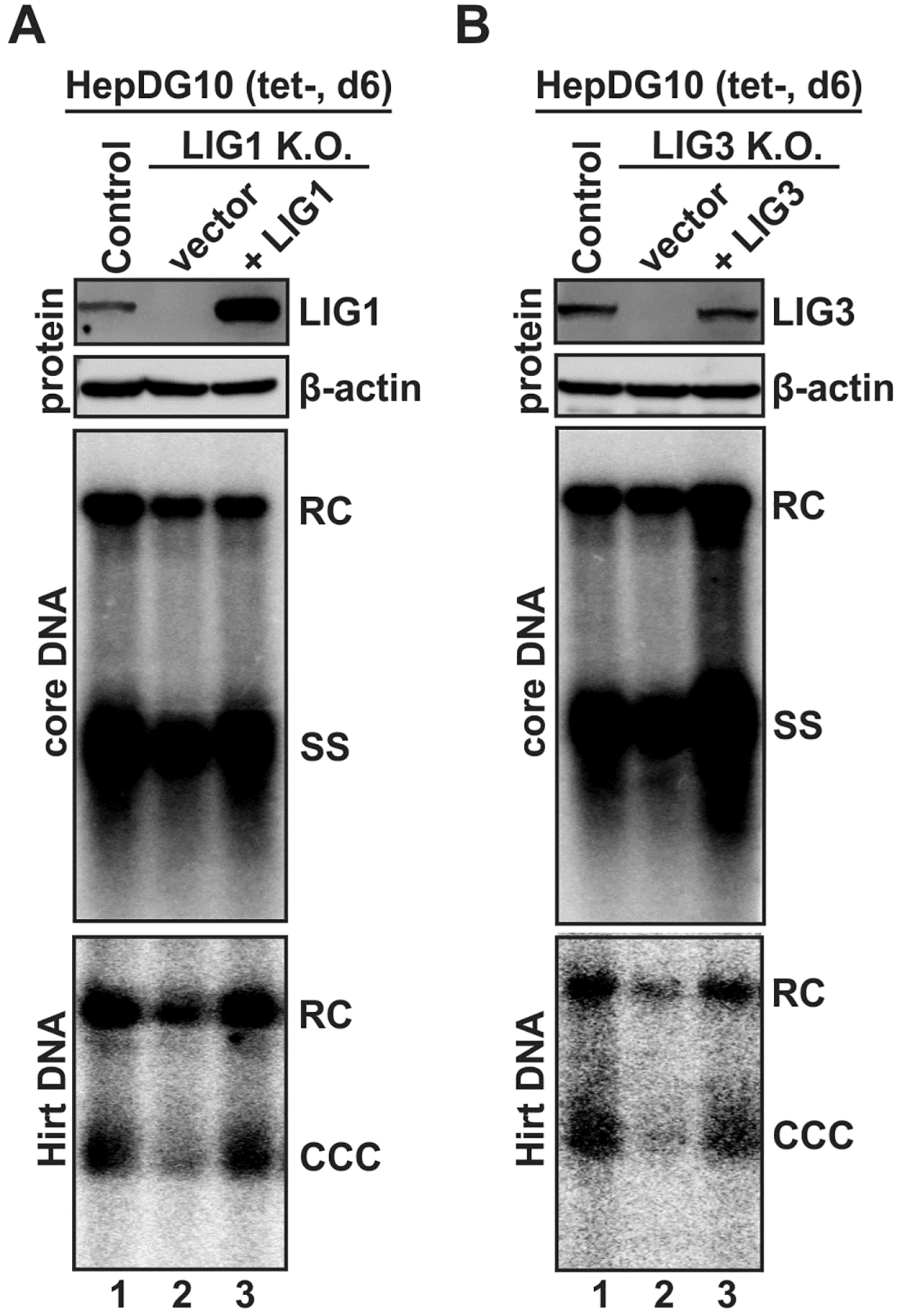
Ectopic expression of LIG1 and LIG3 restored DHBV cccDNA formation in ligase knock-out cells. HepDG10-based control knock-out cells and LIG1 knock-out cells (A) or LIG3 knock-out cells (B) were transfected with control vector or plasmid expressing sgRNA-resistant LIG1 (A) or LIG3 (B), and cultured in tet-free medium to induce DHBV replication. Cells were harvested at day 6 post induction, LIG1 and LIG3 expression levels were detected by Western blot, viral core DNA and cccDNA were analyzed by Southern blot. The loading amount of Hirt DNA was normalized by the relative levels of RC DNA (% of control) on core DNA Southern blot. The presented results are representative of two experimental trials.

In order to further validate the role of DNA ligases in hepadnavirus cccDNA formation and rule out the potential off-target effects caused by CRISPR knock-out, we reconstituted LIG1 and LIG3 expression in their corresponding HepDG10 knock-out cells by transfecting plasmid expressing sgRNA-resistant LIG1 and LIG3 gene, respectively (Fig 7A and 7B, top panels). Upon tet induction, DHBV cccDNA formation was successfully rescued in LIG1 and LIG3 knock-out cells by restoring the expression of each ligase (Fig 7A and 7B, bottom panels), which further confirmed that both LIG1 and LIG3 play a critical and specific role in hepadnavirus cccDNA formation.

Furthermore, LIG1 or LIG3 was ectopically expressed into their corresponding HepG2-NTCP12 shRNA knock-down cells through transfection, followed by HBV infection in the presence of 3TC. As shown in S11 Fig, restoration of LIG1 or LIG3 expression significantly enhanced HBV infection, as visualized by core immunostaining.

### LIG4 is required for hepadnavirus cccDNA formation from the viral double stranded linear DNA genome

**Fig 8:**
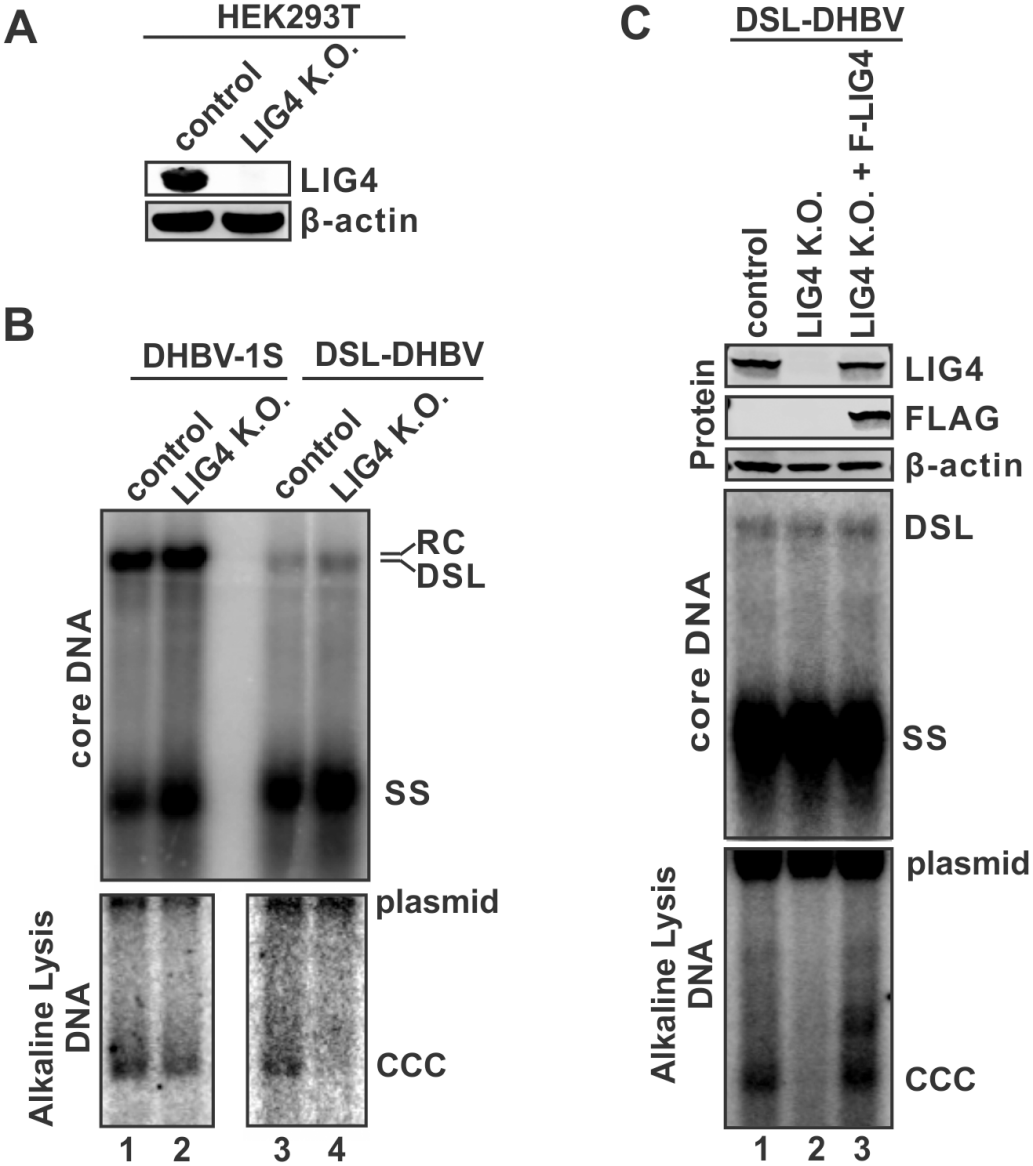
LIG4 is responsible for cccDNA formation from dslDNA. (A) The absence of LIG4 expression in LIG4 knock-out 293T cells was confirmed by Western blot. (B) The control and LIG4 knock-out cells were transfected by plasmid DHBV-1S or DSL-DHBV for 5 days. The intracellular DHBV core DNA and cccDNA were detected by Southern blot. (C) DSL-DHBV was transfected into 293T control knock-out cells and LIG4 knock-out cells with empty vector, or into LIG4 knock-out cells with plasmid expressing FLAG-tagged LIG4, for 5 days. The expression of endogenous and exogenous LIG4 was detected by antibodies against LIG4 or FLAG-tag. The Intracellular DHBV core DNA and cccDNA were analyzed by Southern blot.

With rcDNA serving as the major viral genome DNA form and cccDNA precursor, hepadnavirus replication produces a minor double stranded linear (dsl) DNA species, which can also be converted into cccDNA format [1]. We previously reported that Ku80 protein in cellular NHEJ DNA repair pathway is involved in DHBV cccDNA formation from such dslDNA form [25]. In this study, we set out to assess the role of LIG4, the end effector of NHEJ pathway, in hepadnavirus cccDNA formation through gene knock-out approach. Because the classical CRSIPR/Cas9-based knock-out system requires NHEJ apparatus to introduce indel mutations at the DNA cleavage site, we made use of an alternative microhomology-mediated end-joining (MMEJ) DNA repair based CRISPR PITCh system to obtain the LIG4-null cell line [32] (Materials and methods) (S12 Fig). As shown in Fig 8A, LIG4 expression was completely depleted in the established LIG4 knock-out HEK293T cells. Next, we transfected the control and LIG4 K.O. cells with either the wildtype DHBV-1S construct or a DSL-DHBV plasmid supporting dslDNA-only replication (Fig 8B). The results demonstrated that the cccDNA formation in the context of wildtype DHBV replication was not affected by knocking out LIG4 (Fig 8B, comparing lane 2 to lane 1), while the cccDNA formation from dslDNA was completely abolished in the absence of LIG4 (Fig 8B, lane 4 vs lane 3). Furthermore, restoration of LIG4 expression was able to rescue cccDNA formation in DSL-DHBV transfected LIG4 K.O. cells (Fig 8C). Collectively, the above data strongly supports a conclusion that LIG4 is specifically required for generating cccDNA from the hepadnaviral dslDNA genome.

## Discussion

The establishment and persistence of hepadnavirus infection is dependent upon the viral cccDNA, which is a non-replicating episomal viral genome deposited in the nucleus of infected cell after conformational conversion from viral rcDNA [9]. Due to the limited gene-coding capacity of hepadnavirus genome, the virus needs to borrow host functions to complete its lifecycle [8]. The cellular DNA repair is a well-conserved surveillance and restoration system to detect and heal the damage in chromosomal DNA, by which maintains the stability and integrity of the host genome for replication and transcription [33, 34]. It is plausible that hepadnaviruses hijack the cellular DNA repair apparatus for cccDNA formation by disguising the rcDNA as a “damaged” DNA [9, 20]. The two gaps on rcDNA would be recognized as lesions for DNA repair by the host, and the DNA termini and their associated modifications are expected to undergo trimming, elongation, and ligation, during cccDNA formation (Fig 1). However, the host DNA repair pathway responsible for cccDNA formation remain largely unknown, and thus far, only a few host DNA repair enzymes have been reported to be involved in cccDNA formation, including the tyrosol-DNA phosphodiesterase 2 (TDP2) [17], polymerase κ (POLK) and λ (POLL) [21]. In this study, we screened 107 host DNA repair factors to assess their individual effect on HBV cccDNA formation by lentiviral shRNA knock-down, and identified and validated the host DNA LIG1 and LIG3 as key factors for hepadnavirus cccDNA formation by using a battery of *in vitro* and cell-based assays. Such work hence provides new insights into the mechanisms underlying hepadnavirus cccDNA formation in hepatocyte nucleus.

As part of the cellular DNA replication and repair machineries, DNA ligases complete joining of DNA strands by catalyzing the phosphodiester bond formation. Specifically, LIG1 ligates the Okazaki fragments during chromosomal DNA synthesis, and it is involved in the ligation steps of homologous recombination repair (HRR), long-patch base-excision repair (BER) and nucleotide excision repair (NER); LIG3 is responsible for sealing single strand DNA breaks during the process of short-patch BER and NER [26]. The involvement of DNA ligases in cccDNA formation indicated that the process of rcDNA termini generates ends that can be ligated and DNA ligases are the end-effectors for sealing the breaks of rcDNA. It is worth noting that previous studies have shown that LIG3, but not LIG4, is essential for nuclear DNA replication in the absence of LIG1 [35, 36]; and LIG1 is a backup enzyme for LIG3 in BER and NER DNA repair pathways [37, 38]. Such functional redundancy between LIG1 and LIG3 may explain the unaffected cell viability and the incomplete inhibition of cccDNA formation by knocking down/out LIG1 or LIG3 only (Figs 4A, 5–7 and S8A) or knocking down both (Figs 4B and S8B). In previous studies, the redundant functions in cccDNA formation have also been observed between POLK and POLL [21], and perhaps between TDP2 and an unknown TDP2-like protein(s) [17]. It is of note that the potential role of TDP2 in cccDNA formation remains controversial. While one study demonstrated that knock-down of TDP2 inhibited, or at least delayed, DHBV cccDNA formation [17]; another study suggested that TDP2 might even serve as a negative regulator of HBV cccDNA formation rather than a facilitator [18], and a recent study showed that TDP2 chemical inhibitors did not inhibit HBV infection in cell cultures [39]. In our shRNA screen, POLK or TDP2 lentiviral shRNA did not significantly reduce cccDNA formation in HepDES19 cells (S1 Fig), indicating that cellular functional redundancy for each enzyme might also exist in our experimental system. Further validation and mechanistic studies are required to reconcile these results. On the other hand, it is also possible that the first round cccDNA formation from virion DNA during infection and the intracellular cccDNA amplification pathway may have preference for different DNA repair enzymes, or there is hepatic cell line- or clone-specific requirement of host DNA repair factors/pathways for cccDNA formation. We had attempted to CRIPSR out both ligases in HepDG10 cells but only achieved partial double knock-down (Fig 4B), suggesting that at least one of LIG1 and LIG3 is required by the cells, and perhaps by hepadnaviruses as well. However, our data does not completely rule out a possibility that a LIG1/3-independent ligation mechanism might be involved in cccDNA formation, such as DNA topoisomerase I (TOP1) which has been suggested to play a role in rcDNA circularization through its DNA endonuclease and strand transferase activities [40]. Based on the previous and current data, it can be also inferred that hepadnaviruses have evolved to take advantage of the functional redundancy of host DNA repair machinery for a successful cccDNA formation.

The mechanism underlying the different efficiency of cccDNA formation between HBV and DHBV remains largely unknown, but likely in a virus-specific but not host-specific manner [16, 25, 30]. Based on that, we created the cell-free and the human hepatoma cell-based DHBV system to facilitate the identification and validation of host and viral regulators of cccDNA formation (Figs 2 and 3). In addition to the possible determining factors for cccDNA formation in the steps of rcDNA maturation, deproteinization, nuclear importation and uncoating, whether the cellular DNA repair system differentially recognizes and repairs nuclear HBV and DHBV rcDNA into cccDNA remains obscure. In this study, we found that both viruses employ LIG1 and LIG3 for cccDNA formation (Figs 4–7, S8 and S11), suggesting that the different repair process of HBV and DHBV rcDNA, if any, should be at the steps upstream of rcDNA end joining.

Though LIG1 and LIG3 have overlapping functions, knocking out/down of LIG3 resulted in relatively lower level of cccDNA than LIG1 knock-out/down (Figs 4–7 and S8), which suggests that LIG3 may play a more important role in cccDNA formation. In line with this notion, the two separated nicks/gaps in rcDNA are reminiscent of single strand breaks, which are preferable substrates for LIG3 in BER- and NER-mediated single strand break repair (Fig 1). Moreover, during the primary screen, two other BER components, APEX1 and POLB [41], emerged as candidates for positive regulator of cccDNA formation (S1 Fig), further suggesting the potential involvement of short-patch BER in cccDNA formation, which awaits further systematic investigations.

With the protein and RNA attachments at the 5’ end of minus- and plus-strand, respectively, hepadnavirus rcDNA is not a typical DNA break substrate for the major known repair pathways, and it is unknown whether the two gaps in rcDNA are repaired simultaneously or separately, including the final ligation step. A recent study revealed a nuclear rcDNA species with a covalently closed minus strand but an open plus strand, indicating that the nick on minus strand may be sealed first during cccDNA formation [28]. However, we did not observe an increased accumulation of protein-free rcDNA after blocking cccDNA synthesis in LIG1 or LIG3 knock-out cells (Figs 4, 6, 7B and S8). This phenomena may be due to a fact that the processed rcDNA ready for ligation is unstable. Previous studies have shown that the Hirt DNA samples from DHBV replicating cells contain high levels of nicked cccDNA which might be generated intracellularly or during the Hirt extraction [16, 30]. We also found that the protein-free rcDNA in Hirt extraction from HepDG10 cells were largely nicked cccDNA (S9 Fig), indicating that the observed reduction of protein-free rcDNA in LIG1/3 knock-out cells might be a consequence of cccDNA reduction (Figs 4 and 7). Nonetheless, further characterization of the nuclear rcDNA in LIG1/3 knock-out cells will provide further information for understanding the biological processes of rcDNA termini prior to the final ligation step during cccDNA formation.

In parallel with the *bona fide* rcDNA-to-cccDNA formation during hepadnavirus infection, the viral dslDNA byproduct is also repaired into cccDNA with indel mutations at the joint region [16, 23]. Although the dslDNA-derived cccDNA is generally defective of initiating a new round of viral DNA replication, it remains functional to express HBsAg and thus may play a role in viral pathogenesis. Based on the linear format of dslDNA and the indel mutations of its cccDNA derivative, it is hypothesized that dslDNA is a substrate for host error-prone NHEJ DNA repair system, and we have previously reported that another NHEJ component Ku80 is required for DHBV cccDNA formation from the dslDNA but not rcDNA [25]. LIG4 is the DNA ligase responsible for performing the last step of double strand DNA end joining in the NHEJ pathway [24]. In this study, we have demonstrated that LIG4 plays an essential role in cccDNA formation from DHBV dslDNA, and no functional redundancy was observed between LIG4 and other ligases (Fig 8). In addition, it has been reported that the chromosome DNA double strand breaks are targets for DHBV DNA integration [42], which indicates that the NHEJ machinery, including LIG4, is also responsible for the integration of hepadnavirus dslDNA into host genome.

Altogether, our study revealed a critical role of cellular DNA ligases in hepadnavirus cccDNA biosynthesis. Another possible function of DNA ligases in hepadnavirus life cycle can be to maintain the integrity of cccDNA, provided the preexisting cccDNA undergoes DNA damage and the host cell is able to repair it. Based on our observations, the DNA ligase inhibitors, which are currently under development for anti-cancer therapy [43], may be developed into host-targeting antiviral means to treat chronic hepatitis B by blocking cccDNA formation and/or repair.

## Materials and methods

### Cell lines, viruses, and inhibitors

HepG2 and 293T cells were purchased from ATCC and cultured in DMEM/F12 medium (Gibco) supplemented with 10% fetal bovine serum, 100 U/ml penicillin and 100 μg/ml streptomycin. The tetracycline-inducible HBV (Genbank accession number: U95551) stable cell line HepDE19 and HepDES19 were established previously [15], and maintained in the same way as HepG2, but with the addition of 1 μg/ml tetracycline (tet) and 400 μg/ml G418. When required, the culture medium was switched to tet-free to initiate HBV replication in HepDE19 and HepDES19 cells. HBV infectious particles were collected from the supernatant of HepDE19 cells, and the infection of HepG2-NTCP12 cells and HBV core protein (HBc) immunofluorescence microscopy were conducted according to a previously published protocol [44]. DHBV virions were purified from the serum of virally infected ducks as previously described [19]. DNA ligase 1/3 inhibitors L1 (5-(methylthio)thiophene-2-carboxylic acid) and L25 (2,3-dioxoindoline-7-carboxylic acid), and the pan ligase inhibitor L189 (6-Amino-2,3-dihydro-5-[(phenylmethylene)amino]-2-4(1H)-pyrimidineone) were purchased from Tocris Biosciences. Lamivudine (3TC) was kindly provided by Dr. William Mason (Fox Chase Center Center).

### Hepadnavirus RNA and DNA analyses

DHBV total RNA in cell cultures was extracted by TRIzol (Invitrogen) and detected by Northern blot [25]. HBV and DHBV cytoplasmic core DNA and whole cell Hirt DNA were extracted and analyzed by Southern blot as previously described [15, 45, 46]. HBV total DNA and cccDNA qPCR were performed according to the literature with modifications [47–49]. Firstly, HBV total DNA in the total Hirt DNA sample, including protein-free rcDNA and cccDNA, was first quantified by qPCR with 0.8 μM of forward primer (5’-CCGTCTGTGCCTTCTCATCTG-3’ (nt 1551–1571)), 0.8 μM of reverse primer (5’-AGTCCAAGAGTYCTCTTATGYAAGACCTT-3’ (nt 1674–1646)), and 0.2 μM of TaqMan probe (5’-FAM- CCGTGTGCACTTCGCTTCACCTCTGC-TAMRA-3’ (nt 1577–1602)). In the meantime, the relative cellular mitochondrial DNA (COX3 gene) level in each Hirt DNA samples was quantified by SYBR green qPCR with forward primer 5’-CCCTCTCGGCCCTCCTAATAACCT-3’ and reverse primer 5’-GCCTTCTCGTATAACATCGCGTCA-3’. Next, to reduce the contamination of HBV rcDNA in the qPCR detection of cccDNA, the Hirt DNA sample was first heated at 85°C for 5 min to denature rcDNA into single-stranded DNA, followed by Plasmid-safe ATP-dependent DNase (PSAD) (Epicentre) treatment at 37°C for 16 h. The PSAD reaction was then stopped by heat inactivation at 70°C for 30 min, and the samples were further purified by DNA clean-up spin column (Zymo Research). Real-time PCR amplification of 2 μl cleaned cccDNA sample was performed in a 20 μl reaction containing 0.9 μM forward primer (5’-ATGGAGACCACCGTGAACGCCC-3’(nt 1610–1631)), 0.9 μM reverse primer (5’-TCCCGATACAGAGCTGAGGCGG-3’(nt 2021–2000)), and 0.2 μM TaqMan probe (5’-FAM-TTCAAGCCTCCAAGCTGTGCCTTGGGTGGC-TAMRA-3’; nt 1865–1894). The cccDNA qPCR primers were designed to target the HBV DNA sequences outside of the gap region in rcDNA and to avoid PCR amplification of the integrated HBV genome in HepDES19 cells. The FastStart Essential DNA Probes Master (Roche) and FastStart Universal SYBR Green Master (Roche) were used to assemble TaqMan and SYBR Green qPCR reactions, respectively. The qPCR was run by Roche LightCycler 96 under the following thermal cycling conditions: 10 min at 95°C, followed by 15 sec at 95°C and 1 min at 61°C for 50 cycles. The cccDNA qPCR data was normalized by the cellular mitochondrial DNA quantitation.

The supercoiled cccDNA from DHBV transfected 293T cells were extracted by a previously developed alkaline lysis method with minor modifications [22]. Briefly, cells in a 6-well-plate were lysed in 200 μl lysis buffer containing 10 mM Tris-HCl (pH7.4), 1 mM EDTA, and 0.2% NP40, at room temperature for 10 min. The lysate was mixed gently with 200 μl alkaline lysis buffer (0.1 M NaOH, 6% SDS) and incubated at 37°C for 30 min, followed by adding 100 μl 3 M KAc (pH5.0) and mixing gently. After incubating on ice for 10 min and centrifuging at 12,000 rpm for 5 min, supernatant was collected and extracted by phenol twice. DNA was precipitated by ethanol and subjected to Southern blot assay.

### shRNA screen

The customized Mission lentiviral shRNA DNA repair gene family set was purchased from Sigma-Aldrich. The library contains 586 lentiviral shRNA targeting 140 DNA repair genes of all the known DNA repair pathways except for NHEJ, with an average of 3–5 different shRNA sequence against each target gene. The virus stocks were aliquoted in 96-well-plate with viral titer ranging from 1×10^7^ to 3×10^7^ g.e/ml. The TDP2 lentiviral shRNA was purchased from Santa Cruz Biotechnology and added into the library.

To establish stable DNA repair gene knock-down cell lines. HepDES19 cells were infected with the pooled lentiviral shRNA targeting the same DNA repair gene or control lentiviral shRNA in the presence of tet, 2 days later, the cells were selected by puromycin (3 μg/ml) for 1 week and the antibiotics-resistant cells were pooled and expanded into cell lines. The cells transduced by different lentiviral shRNA exhibited variable growth rate during puromycin selection but all were viable. A total of 107 DNA repair gene lenti-shRNA transduced cell lines were obtained. To assess the effect of RNAi on cccDNA production, the knock-down cells under confluent condition (1×10^6^ cells per 35mm-dish) were cultured in the absence of tet for 10 days, total Hirt DNA was extracted and subjected to Southern blot or HBV total DNA and cccDNA qPCR. It is known that the HBV Hirt DNA level is positively related to HBV DNA replication level, especially the cytoplasmic rcDNA level [15, 16, 19]. To assess the efficiency of cccDNA formation under gene knock-down, the relative cccDNA levels in DNA repair gene knock-down cells compared to control knock-down cells were normalized by total HBV Hirt DNA signals, which indicates a relative rcDNA-to-cccDNA conversion rate.

### Plasmids

Plasmid pTREHBVDE, the vector delivered the HBV transgene in HepDE19 cells, has been described previously [15]. DHBV-1S is a plasmid supporting DHBV (Genbank Accession No.: K01834) DNA replication upon transfection into cell cultures [50]. Plasmid 1Sdsl-3 (renamed to DSL-DHBV in this study) was a derivative of DHBV-1S with an artificial point mutation of G2552C in viral genome that supports double stranded linear (dsl) DNA replication but fails to make rcDNA [25, 51]. Plasmid pcLIG1-FLAG expressing human DNA ligase 1 (LIG1, GenBank Accession No.: NM_000234) with C-terminal FLAG-tag was purchased from Genescript (clone ID: OHu14319). To construct ligase 3 (LIG3) expression plasmid pcLIG3, the ORF of LIG3 was PCR amplified from MGC human LIG3 sequence-verified cDNA (GE Healthcare Dharmacon, clone ID: 6092747) by forward primer 5’-5’-CGGGATCCATGTCTTTGGCTTTCAAGATCTTCTT-3’ (BamHI site is underlined) and reverse primer 5’-GCTCTAGACTAGCAGGGAGCTACCAGTCTCCGTTT-3’ (XbaI site is underlined), and cloned into the BamHI/XbaI restricted pcDNA3.1 vector (Invitrogen). Plasmid pcLIG4-FLAG expressing the C-terminal FLAG-tagged human Ligase 4 (LIG4) was purchased from Genescript (clone ID: OHu13291).

### In vitro cccDNA formation in nuclear extract

DHBV virion DNA which contain predominantly rcDNA and a minor portion of dslDNA were purified from serum derived virions and quantified by Southern blot using DHBV DNA marker as standard according to published literature [52]. DHBV cccDNA was extracted from Dstet5 cells and gel purified as previously described [15]. The nuclear extract was prepared from HepG2 cells and stored in aliquots following a published protocol [53]. To assemble the DNA repair reaction, the purified DHBV virion DNA was mixed with 4 μl nuclear extract in 200 μl reaction buffer containing 20 mM HEPES, 80 mM KCl, 10 mM MgCl_2_, 1 mM ATP, 1 mM DTT, and 50 μM dNTPs, and incubated at 37°C for 30 min. To stop the reaction, 10 μl of 1% SDS, 20 μl of 0.5 M EDTA and 10 μl of 10 mg/ml pronase were added and incubated at 37°C for an additional 30 min. Next, the mixture was subjected to phenol and phenol:chloroform extraction, and viral DNA was precipitated down by ethanol and dissolved in 10 μl nuclease-free H2O. The obtained viral DNA was analyzed by Southern blot or cccDNA-specific PCR. The PCR reaction was assembled by mixing 0.5 μl DNA sample, 12.5 μl 2× PCR buffer (Clontech), 1 μl of 20 μM forward primer (5’-GCCAAGATAATGATTAAACCACG-3’), 1 μl of 20 μM reverse primer (5’-TCATACACATTGGCTAAGGCTC-3’), 0.5 μl Terra polymerase (Clontech), and 9.5 μl H2O. The DNA was amplified by 22 cycles of heat denaturation at 95°C for 30 sec, annealing at 55°C for 30 sec, and extension at 72°C for 30 sec. The PCR product was subjected to agarose gel electrophoresis and stained by ethidium bromide.

### Treatment of rcDNA by Exo I and Exo III

To detect the possible closed minus strand or plus strand after *in vitro* cccDNA formation in nuclear extract, exonucleases Exo I and III (ExoI/III) were used to degrade DNA strands with a free 3’ end and preserve closed circular DNA in either single-stranded (SS) or double-stranded (DS) form as described previously [28]. Briefly, 5 ng of DHBV rcDNA were subjected to the *in vitro* cccDNA formation reaction as described above. After reaction, the recovered DNA were dissolved in 20 μl water and treated with 0.25 μl each of Exo I and Exo III at 37°C for 2 h in 1×NEB Cutsmart buffer. 5 ng of DHBV rcDNA without going through *in vitro* cccDNA formation reaction served as positive control for ExoI/III digestion. The digestion products were directly subjected to electrophoresis and Southern blotting for hybridization with p32-labeled DHBV minus- or plus-strand specific riboprobe.

### Establishment of HepDG10 cell line

Plasmid pTRE-GFP-DHBV, which bidirectionally supports the tet-inducible expression of GFP and DHBV pgRNA, was constructed as follows. Firstly, a DNA fragment, which, in the 5’ to 3’ orientation, contained the partial sequence (nt 12–430) of pBI vector (Clontech, GenBank Accession No.: U89932) and the reverse complementary sequence of GFP ORF followed by SV40 polyadenylation signal, with unique PstI and KpnI restriction site at the 5’ and 3’ end, respectively, was chemically synthesized (Genescript). Then the PstI/KpnI restricted fragment was cloned into the same endonuclease treated pTREHBVDE plasmid to generate pTRE-GFP-HBV. Next, another DNA fragment containing nt 425–468 sequence from pBI, a spacer sequence (5’-GCAGAGCTCGTTTGATC-3’), and DHBV sequence (nt 2524-3021/1), with unique KpnI and EcoRI site at 5’ and 3’ end, respectively, was chemically synthesized (Genescript), and the fragment was inserted into the KpnI/EcoRI sites of pTRE-GFP-HBV to generate pTRE-GFP-DHBV-EcoRI-HBV. The HindIII site in the backbone sequence downstream of the remaining HBV sequence in pTRE-GFP-DHBV-EcoRI-HBV was further mutated to SalI site by using QuikChange II Site-Directed Mutagenesis Kit (Agilent Technologies) to obtain pTRE-GFP-DHBV-EcoRI-HBV-SalI. One unit length of DHBV genome was amplified from DHBV-1S by PCR with forward primer (5’- CGGCTAGAATTCATGCTCATTTGAAAGCTT-3’, nt 3011-3021/1-19, DHBV EcoRI site is underlined) and reverse primer (5’-AATTAAGTCGACAATTCTAGCCGTAATCGGATA-3’, nt 3021–3001, non-DHBV SalI site is underlined) and digested by EcoRI and SalI and cloned into the same sites in pTRE-GFP-DHBV-EcoRI-HBV-SalI to generate the final product pTRE-GFP-DHBV.

To establish tetracycline-inducible DHBV stable cell lines, HepG2 cells were cotransfected by pTRE-GFP-DHBV and pTet-off which expresses tet-responsive transcriptional activator (tTA) (Clontech) with 7:1 molar ratio. The transfected HepG2 cells were selected with 500 μg/ml G418 in the presence of 1 μg/ml tet. G418-resistant colonies were picked and expanded into cell lines. To determine DHBV positive cell lines, the candidate clones were cultured in 96-well-plate with tet-free medium for 6 days and subjected to fluorescence microscopy to select GFP-positive clones. Then, the GFP-positive cells were lysed in 1% NP40 and the cytoplasmic lysate was subjected to dot blotting as described previously [47]. The dot blot was hybridized by α-^32^P-UTP (800 Ci/mmol, Perkin Elmer) labeled minus strand specific full-length DHBV riboprobe, and the obtained DHBV positive cell lines were further assessed for their tet-inducible DHBV core DNA replication and cccDNA production by Southern blot. A DHBV cccDNA highly producing cell line clone was named HepDG10. The maintenance and induction of HepDG10 cells were performed in the same way as HepDES19 cells.

### Establishment of LIG1- and LIG3-knock-down cell lines

Lentiviral particles expressing U6 promoter-driven shRNA for knocking down human LIG1 or LIG3 were purchased from Sigma-Aldrich. The shRNA coding sequences for knocking down LIG1 and LIG3 are listed in S1 Table. HepDG10 and HepG2-NTCP12 cells were transduced by above lentiviral LIG1 or LIG3 shRNA or control shRNA per manufacturer’s instruction. The transduced cells were selected with 3 μg/ml puromycin and the antibiotics-resistant cells were pooled and expanded into cell lines. The knock-down levels of LIG1 and LIG3 in knock-down cell lines were assessed by Western blot by using antibodies against LIG1 (sc-271678, clone C5, Santa Cruz Biotechnology) and LIG3 (sc-390922, clone E7, Santa Cruz Biotechnology), respectively, and compared to control knock-down cells. β-actin served as loading control for Western blot by using anti-actin antibody (MAB1501, clone C4, Millipore).

To construct a lenti-vector expressing both LIG1 and LIG3 shRNA, a chemically synthesized DNA fragment containing the H1 promoter sequence and downstream LIG3 shRNA sequence (S1 Table) was cloned into the unique EcoRI site of lenti-shRNA plasmid DNA pLKO.1-LIG1 (TRCN0000048494, Sigma), giving rise to the bicistronic lenti-shRNA plasmids with two orientations of the H1-shLIG3 cassette right downstream of the original U6-shLIG1 cassette. The head-to-tail and tail-to-tail dimer clones were named pLKO.1-LIG1/3C1 and pLKO.1-LIG1/3C2, respectively, and pLKO.1-LIG1/3C2 was used to prepare lentiviral shRNA particles with MISSION Lentiviral Packaging Mix (Sigma). HepDG10 cells were transduced with lentiviral shLIG1/3 and the puromycin-resistant cells were pooled and expanded into stable cell line, namely HepDG10-shLIG1/3. The double knock-down of LIG1 and LIG3 were determined by Western blot comparing to the aforementioned control knock-down cells.

### Establishment of LIG1- and LIG3-knock-out cell lines

LIG1 and LIG3 knock-out cell lines were generated through CRISPR-mediated genome editing of LIG1 and LIG3 gene loci. The single guide (sg) RNAs targeting two different sites of human LIG1 and LIG3 gene were designed at http://www.e-crisp.org/E-CRISP and shown in S5 and S6 Figs. In addition to the general criteria for sgRNA design [54], the sgRNAs were designed to target either the 5’ end of the ORF or the functional domain coding sequences of LIG1 or LIG3. Furthermore, the designed sgRNA sequences do not possess any possible CRISPR sites in DHBV or HBV sequences. The synthetic sgRNA oligo pairs (S2 Table) were annealed and cloned into BbsI-digested lentiCRISPRv2 control vector (Addgene # 52961, gift from Dr. Feng Zhang). Lentivirus preparations were performed according to the protocols from Dr. Feng Zhang’s Lab (genome-engineering.org). Briefly, each lentivector was co-transfected with packaging plasmids psPAX2 and pMD2.G (Addgene# 12260 and 12259, respectively, gift from Dr. Didier Trono) in molar ratio of 4:3:1 into 293T cells by Lipofectamine 2000 (Invitrogene), and 48 h later, media was collected, centrifuged at 1,000 × g for 10 min, filtered through a 0.45um filter, and virus titers were determined by lentiviral titration kit. Lentiviral transduction of HepDG10 or HepDES19 and antibiotics-selection were performed as above described to generate control and LIG1/LIG3 stable knock-out cell lines, specifically HepDG10 LIG1 K.O., HepDG10 LIG3 K.O., HepDES19 LIG1 K.O., and HepDES19 LIG3 K.O. cells. The LIG1 or LIG3 knock-out phenotype was confirmed by Western blot and indel sequencing assay. The corresponding coding sequences for epitopes of LIG1 and LIG3 antibodies have no overlap with the gene targeting sites of the designed sgRNAs.

To knock out both LIG1 and LIG 3 in HepDG10 cells, the cells were transduced with lentiviruses encoding CRISPR/Cas9-LIG1-sgRNA1 and CRISPR/Cas9-LIG3-sgRNA1 together (1:1 ratio). The puromycin selection and clone screening was performed as described above. The indel mutations of sgRNA targeting sites in LIG1 and LIG3 gene loci was detected by T7E1 assay.

### Indel detection

For indel sequencing analysis of LIG1 and LIG3 genes, total genomic DNA from the control and LIG1 or LIG3 knock-out cells were extracted using DNeasy blood and tissue kit (Qiagen) according to the manufacturer’s protocol. The genomic sequence region covering the CRISPR target site was amplified by PCR using the indel detection primers (S3 Table) and cloned into T vector pMD19 (Clontech) for Sanger sequencing. The LIG1/3 DNA sequence from control and knock-out cells was aligned to determine the CRISPR-induced mutations.

For T7E1-based indel assay, primers used to amplify DNA fragments containing LIG1-sgRNA1 and LIG3-sgRNA1 targeting region were listed in S3 Table, indel mutations were detected by Guide-it Mutation Detection Kit (Clontech) according to manufacturer’s manual.

### Generation of LIG4 knock-out cell line

Because LIG4 is an essential component in NHEJ DNA repair pathway in eukaryotes [55, 56], the conventional NHEJ-based CRISPR/Cas9 knock-out system is not able to generate LIG4 knock-out cell lines. Therefore a newly developed microhomology-mediated end-joining (MMEJ) repair based CRISPR/Cas9 knock-in system [32] was used to establish LIG4 knock-out cells. Briefly, annealed sgRNA targeting the last exon of LIG4 gene (S12 Fig and S4 Table) was inserted into pX330A-1×2 (Addgene# 58766, gift from Dr. Takashi Yamamoto) to obtain pX330A-1×2-LIG4-gRNA, and after Golden Gate assembly using BsaI (New England Biolabs), the cassette of PITCh-gRNA from pX330S-2-PITCh (Addgene# 63670, gift from Dr. Takashi Yamamoto) was inserted into pX330A-1×2-LIG4-gRNA to generate the All-in-One pCRISPR/Cas9-PITCh-LIG4-gRNA vector containing both LIG4 gRNA and PITCh-gRNA. Then, pCRIS-PITChv2-LIG4 was constructed based on pCRIS-PITChv2-FBL (Addgene# 63672, gift from Dr. Takashi Yamamoto) by performing two separate PCR, one to amplify the vector backbone and one to amplify LIG4-specific microhomology arm containing EGFP-2A-Puro knock-in cassette. Generic 5′-reverse and 3′-forward primers were used for vector backbone amplification, and LIG4-specifc primers containing the desired microhomologies were used to amplify the insertion (S4 Table). The above two purified PCR fragments were conjugated by using the In-Fusion HD cloning kit (Clontech) to generate plasmid pCRIS-PITChv2-LIG4. To generate LIG4 knock-out cell line, 293T cells were transfected with pCRIS-PITChv2-LIG4 and the All-in-One plasmid pCRISPR/Cas9-PITCh-LIG4-gRNA with a molar ratio of 1:2, followed by puromycin (1 μg/ml) selection. The puromycin-resistant colonies were pooled together and subjected to fluorescence microscopy for determining EGFP-positive cells with successful knock-in, and then the knock-out efficiency of LIG4 was determined by Western blot using antibodies against LIG4 (sc-28232, clone H-300, Santa Cruz Biotechnology). Control knock-in 293T cells were made by transfecting pCRIS-PITChv2-FBL and pX330A-FBL/PITCh (Addgene# 63671, gift from Dr. Takashi Yamamoto) that target human fibrillarin (FBL) gene.

### Ectopic expression of LIG1, LIG3, and LIG4 in knock-out cells

The “NGG” protospacer adjacent motif (PAM) sequence of LIG1 sgRNA1 locates just ahead of the targeted exon (S5 Fig), therefore plasmid pcLIG1-FLAG was directly used in the function rescue experiment by transfecting the HepDG10-LIG1 K.O. cells. To avoid the integrated lentiviral CRISPR/Cas9-LIG3 sgRNA system targets LIG3 ectopic expression plasmid, the LIG3 sgRNA1 corresponding PAM motif “CGG” in pcLIG3 was mutated to “CGA” by Q5 Site-Directed Mutagenesis Kit (New England Biolabs) with primers (forward: 5’-AACTAGAGCGaGCCCGGGCCA-3’, reverse: 5’- TCTCAAACATGCATTTAATGTGGTACCAC-3’) but without changing the amino acid sequence of LIG3. The sgRNA-resistant pcLIG3 was used to transfect HepDG10-LIG3 K.O. cells in the function rescue experiment. Because the LIG4 knock-out 293T cells were made by transient transfection-mediated knock-out, pcLIG4-FLAG was used directly in rescue experiment by transfection.

## Supporting information

S1 Fig Lenti-shRNA screen of cellular DNA repair genes involved in HBV cccDNA formation. HepDES19 cells were stably transduced with control lentiviral shRNA or lentiviral shRNA targeting different cellular DNA repair genes. After HBV DNA replication and cccDNA formation were induced by tet withdrawal for 10 days, total Hirt DNA extracted from the cells was analyzed by HBV DNA Southern blot and/or qPCR, the relative cccDNA levels in control and DNA repair gene knock-down cells were normalized by total Hirt DNA levels and plotted. The results were grouped according to the known DNA repair pathways where the indicated DNA repair genes primarily belong to (HRR: homologous recombination repair; NER: nucleotide excision repair; BER: base excision repair; DSB: double strand break repair; DDS: DNA damage signaling; cell cycle related; and others). Those 8 genes showed more than 50% reduction of cccDNA upon knock-down were marked by asterisk.
(TIF)

S2 Fig DHBV cccDNA-specific PCR. (A) Schematic illustration of DHBV cccDNA and rcDNA. A pair of primers (P1: nt 2687–2666; P2: nt 2276–2298) crossing the gap region on rcDNA was used for cccDNA-specific PCR. (B) Validation of the sensitivity and specificity of DHBV cccDNA PCR assay. The indicated amount of purified DHBV cccDNA from Dstet5 cells and rcDNA from duck serum were subjected to cccDNA-specific PCR for 25 PCR cycles. (C) Further validation of DHBV cccDNA PCR. 0.3 pg of cccDNA was compared with excess amount of rcDNA in cccDNA-specific PCR reaction under 22 PCR cycles.
(TIF)

S3 Fig ExoI/III treatment of DHBV DNA after *in vitro* cccDNA formation assay. DHBV rcDNA (5 ng) were left untreated (lane 1) or treated with ExoI/III (lane 2) as described in Materials and Methods. In addition, 5 ng of DHBV rcDNA were subjected to the *in vitro* cccDNA formation reaction and the recovered DNA were left undigested (lane 3) or digested by ExoI/III (lane 4). The digestion products were analyzed by Southern blot hybridization with DHBV minus- or plus-strand specific riboprobe.
(TIF)

S4 Fig Validation of cccDNA extracted from HepDG10 cells. cccDNA produced in HepDG10 cells at day 10 post induction was extracted by Hirt extraction and subjected to Southern blot hybridization. To further validate the authenticity of DHBV cccDNA, Hirt DNA samples were heated to 85°C for 5 min before gel loading, a condition that denatures rcDNA into ssDNA, while the cccDNA stays undenatured and its electrophoretic mobility remains unchanged (lanes 1–2). The heat denatured DNA samples were further digested with EcoRI, by which the cccDNA was linearized to double-stranded DNA (lane 3).
(TIF)

S5 Fig LIG1 knock-out by CRISPR/Cas9 DNA editing tool and indel sequencing. (A) Schematic illustration of LIG1 gene locus. The green boxes indicate exons and the solid lines indicate introns. Two designed sgRNA are shown in nucleotide sequence (green) with adjacent PAM sequence shown in red, and their corresponding targeting sites in LIG1 gene are marked. sgRNA1 and sgRNA2 were used to CRISPR out LIG1 in HepDG10 and HepDES19 cells, respectively. The procedure for making LIG1 knock-out cells by CRISPR/Cas9 system is described in Materials and Methods. (B) Indel sequencing assay of HepDG10 cells with LIG1 knock-out. DNA fragment spanning the sgRNA1 targeting region of LIG1 gene loci was PCR amplified from the control and LIG1 knock-out HepDG10 cells and sequenced. The nucleotide sequences of wildtype and CRISPR-mutated LIG1 gene fragment are aligned. The nucleotides within exons are underlined. The omitted sequence of the intron was indicated with a solid line. The deletion mutations of CRISPRed LIG1 gene is shown as dashed line. The mutation-mediated disruption and frame shift of LIG1 ORF are depicted with amino acid codes. (C) Indel sequencing assay of LIG1 deficient HepDES19 cells. Alignment of the sgRNA2 targeting region in LIG1 gene loci from the control and LIG1 knock-out HepDES19 cells shows indels, frameshift, and premature termination of LIG1 ORF in the knock-out cells.
(TIF)

S6 Fig LIG3 knock-out and indel assay. (A) Schematic illustration of LIG3 gene locus, exons are shown in green rectangles. The designed sgRNAs with PAM motif are shown in nucleotide sequence. (B) Indel sequencing analysis of HepDG10 LIG3 knock-out cells. Compared to the wildtype LIG3 sequence in HepDG10 control knock-out cells, a stop codon was introduced into the exon1 of LIG3 gene loci in HepDG10 LIG3 knock-out cells by sgRNA1-guided CRISPR editing. (C) Indel sequencing analysis of HepDES19 LIG3 knock-out cells. The sequencing result demonstrated a frameshift mutation of LIG3 ORF in HepDES19 LIG3 knock-out cells.
(TIF)

S7 Fig T7E1 indel assay of the HepDG10-LIG1/3 K.D. cells. (A) Schematic illustration of the PCR amplicons of LIG1 and LIG3 DNA fragments containing the corresponding sgRNA targeting region. The sequences of sgRNA and PCR primers are listed in S2 and S3 Tables, respectively. (B) The DNA fragments were PCR amplified from genomic DNA of HepDG10-LIG1/3 K.D. cells transduced by CRISPR/Cas9-LIG1/3 sgRNA, the indel mutations were detected by T7E1 digestion.
(TIF)

S8 Fig Knock-down of LIG1/3 reduced cccDNA formation in HepDG10 cells. HepDG10 control and ligase single knock-down cells (A) and double knock-down cells (B) were cultured in tet-free medium for the indicated duration. The levels of LIG1 and LIG3 in each cell lines were determined by Western blot with β-actin serving as loading control. DHBV core DNA and cccDNA were analyzed by Southern blot. The loading amount of Hirt DNA was normalized based on the relative levels of RC DNA (% of control) on core DNA Southern blot.
(TIF)

S9 Fig The protein-free rcDNA in Hirt DNA from HepDG10 cells is predominantly nicked cccDNA. (A) Schematic illustration of the conformational change of true rcDNA and nicked cccDNA in response to mild heat treatment. When heating up to 68°C, the cohesive end of rcDNA between two single strand gaps is melted and the rcDNA is converted into dslDNA, while the nicked cccDNA remains resistant to heat. (B) The cytoplasmic DHBV core DNA and whole cell Hirt DNA extracted from HepDG10 cells (tet-, d6) were left untreated or heated under 68°C for 15 min, followed by Southern blot analysis.
(TIF)

S10 Fig Elimination of RC DNA from Hirt DNA sample. Hirt DNA extracted from HepDES19 cells cultured in tet-free medium was directly subjected to electrophoresis (lane 1), or pretreated by heat denaturation at 85°C for 5 min before gel loading (lane 2), or digested by PSAD with or without heat denaturation prior to electrophoresis (lanes 3 and 4). The HBV DNA signals were detected by Southern blot.
(TIF)

S11 Fig Ectopic expression of LIG1 or LIG3 rescued HBV infection in HepG2-NTCP12 knock-down cells. HepG2-NTCP12 shControl (shCtrl), shLIG1, and shLIG3 cells were transfected with empty vector, or plasmid expressing wild-type LIG1 or LIG3, as indicated. Followed by HBV infection in the presence of 10 μM 3TC for 6 days. (A) The expression levels of LIG1 and LIG3 were determined by Western blot. β-actin served as loading control. (B) The intracellular core protein (HBc) expression was detected by immunofluorescence microscopy. Each image shown here represents five different microscopic fields, the percentage of HBc positive cells was indicated (Mean ± SD).
(TIF)

S12 Fig Schematic illustration of MMEJ-based CRISPR knock-out of LIG4 gene. The exons of LIG4 gene locus are shown in green boxes. The sequence of LIG4 sgRNA is shown and its targeting site in exon 3 of LIG4 is marked. The MMEJ-based CRISPR PITCh system will knock in an EGFP-2A-Puro cassette into the sgRNA targeting site of LIG4 ORF to disrupt gene expression. Refer to Materials and Methods for detailed experimental procedures.
(TIF)

S1 Table shRNA sequence for knock-down of human LIG1 and LIG3. (PDF)

S2 Table Oligos for LIG1/3 CRISPR sgRNA. (PDF)

S3 Table Oligos for LIG1/3 sgRNA indel sequencing and T7E1 assay. (PDF)

S4 Table Oligos for LIG4 sgRNA and knock-in. (PDF)
