# Supplementary figures and images for: The role of host DNA ligases in hepadnavirus covalently closed circular DNA formation
Source: PLoS Pathog. 2017 Dec 29;13(12):e1006784. doi: 10.1371/journal.ppat.1006784 (PMC5747486; doi:10.1371/journal.ppat.1006784)

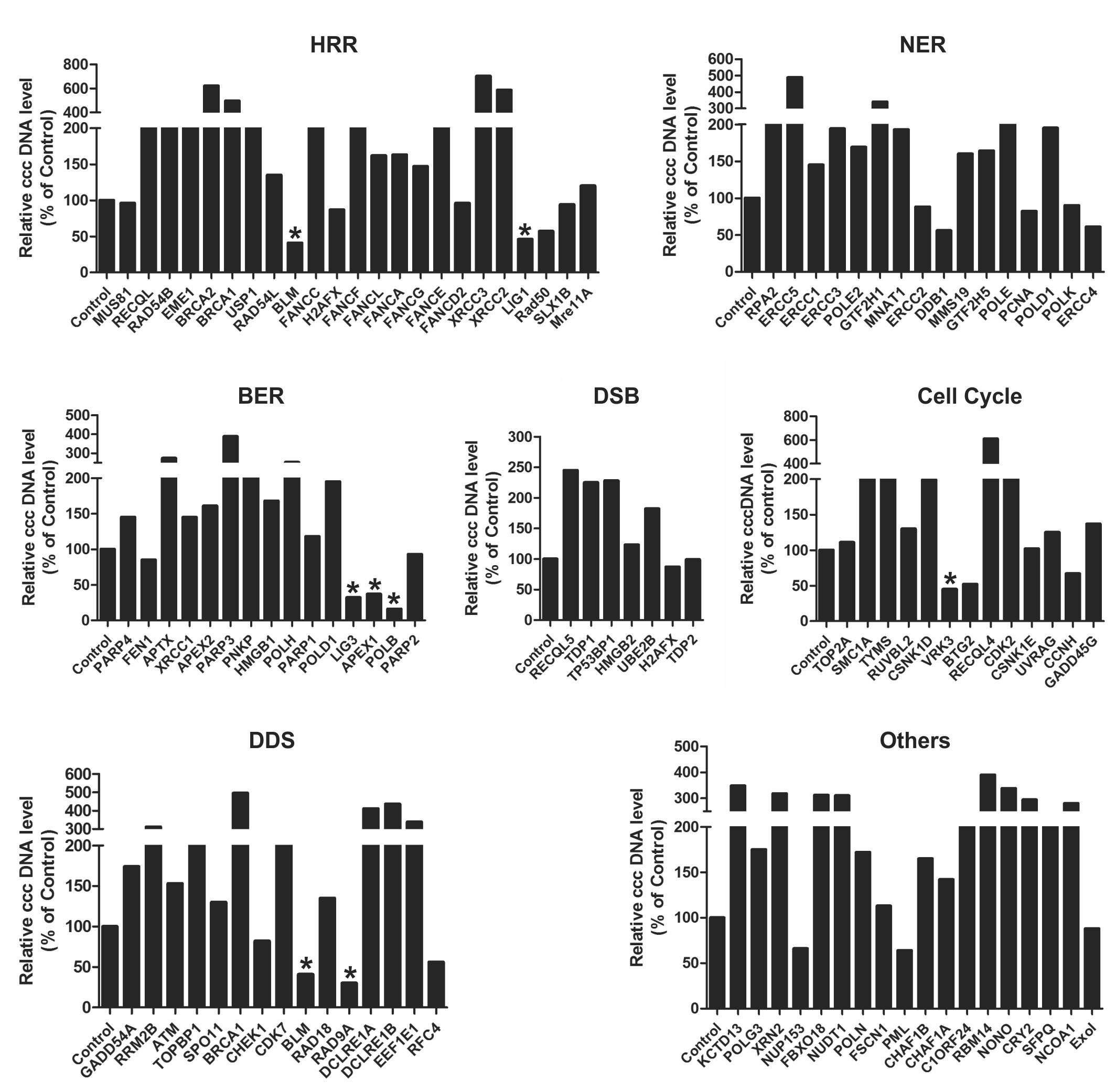

Supplement: S1 Fig — HepDES19 cells were stably transduced with control lentiviral shRNA or lentiviral shRNA targeting different cellular DNA repair genes. After HBV DNA replication and cccDNA formation were induced by tet withdrawal for 10 days, total Hirt DNA extracted from the cells was analyzed by HBV DNA Southern blot and/or qPCR, the relative cccDNA levels in control and DNA repair gene knock-down cells were normalized by total Hirt DNA levels and plotted. The results were grouped according to the known DNA repair pathways where the indicated DNA repair genes primarily belong to (HRR: homologous recombination repair; NER: nucleotide excision repair; BER: base excision repair; DSB: double strand break repair; DDS: DNA damage signaling; cell cycle related; and others). Those 8 genes showed more than 50% reduction of cccDNA upon knock-down were marked by asterisk. (TIF) [file ppat.1006784.s001.tif]

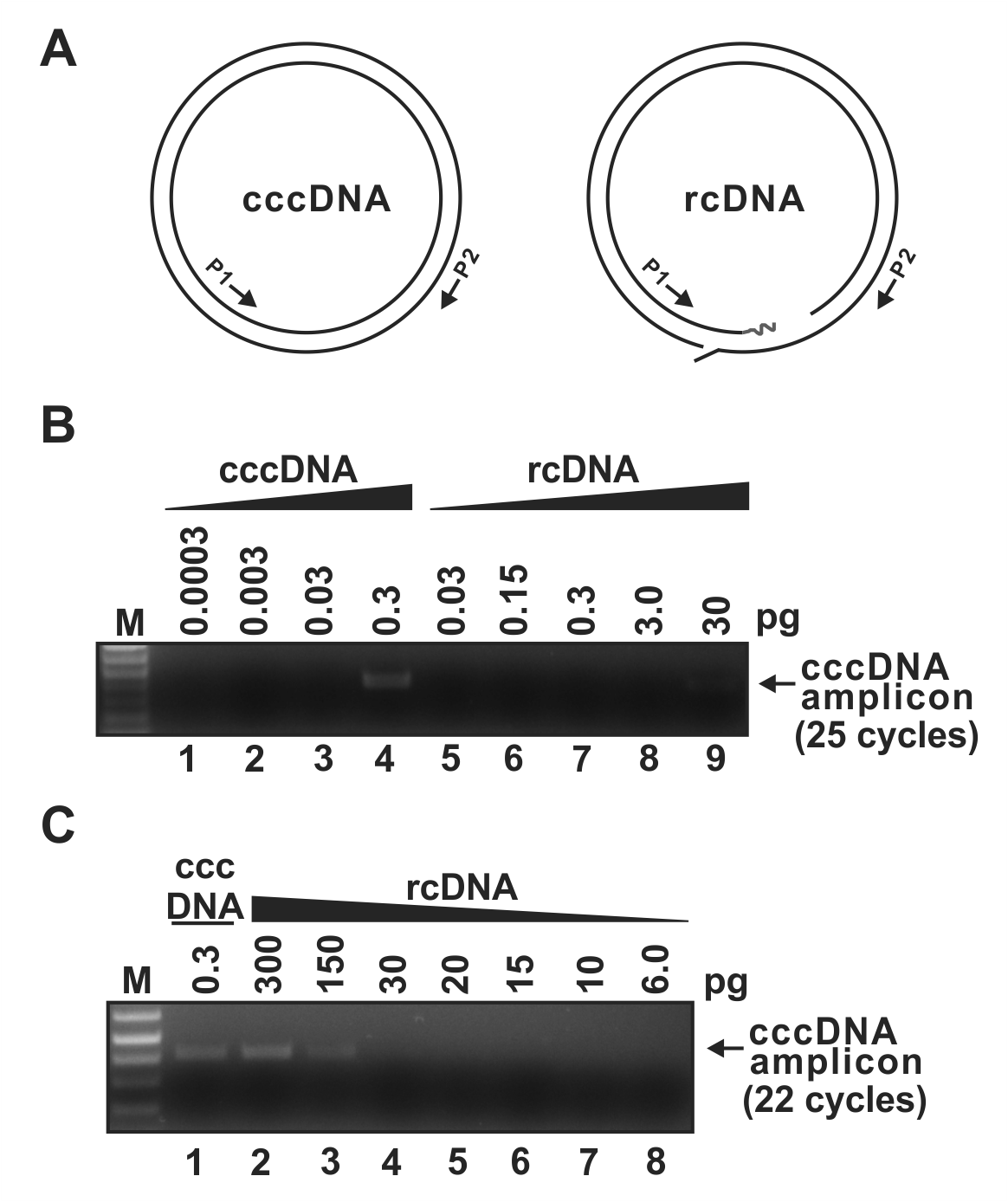

Supplement: S2 Fig — (A) Schematic illustration of DHBV cccDNA and rcDNA. A pair of primers (P1: nt 2687–2666; P2: nt 2276–2298) crossing the gap region on rcDNA was used for cccDNA-specific PCR. (B) Validation of the sensitivity and specificity of DHBV cccDNA PCR assay. The indicated amount of purified DHBV cccDNA from Dstet5 cells and rcDNA from duck serum were subjected to cccDNA-specific PCR for 25 PCR cycles. (C) Further validation of DHBV cccDNA PCR. 0.3 pg of cccDNA was compared with excess amount of rcDNA in cccDNA-specific PCR reaction under 22 PCR cycles. (TIF) [file ppat.1006784.s002.tif]

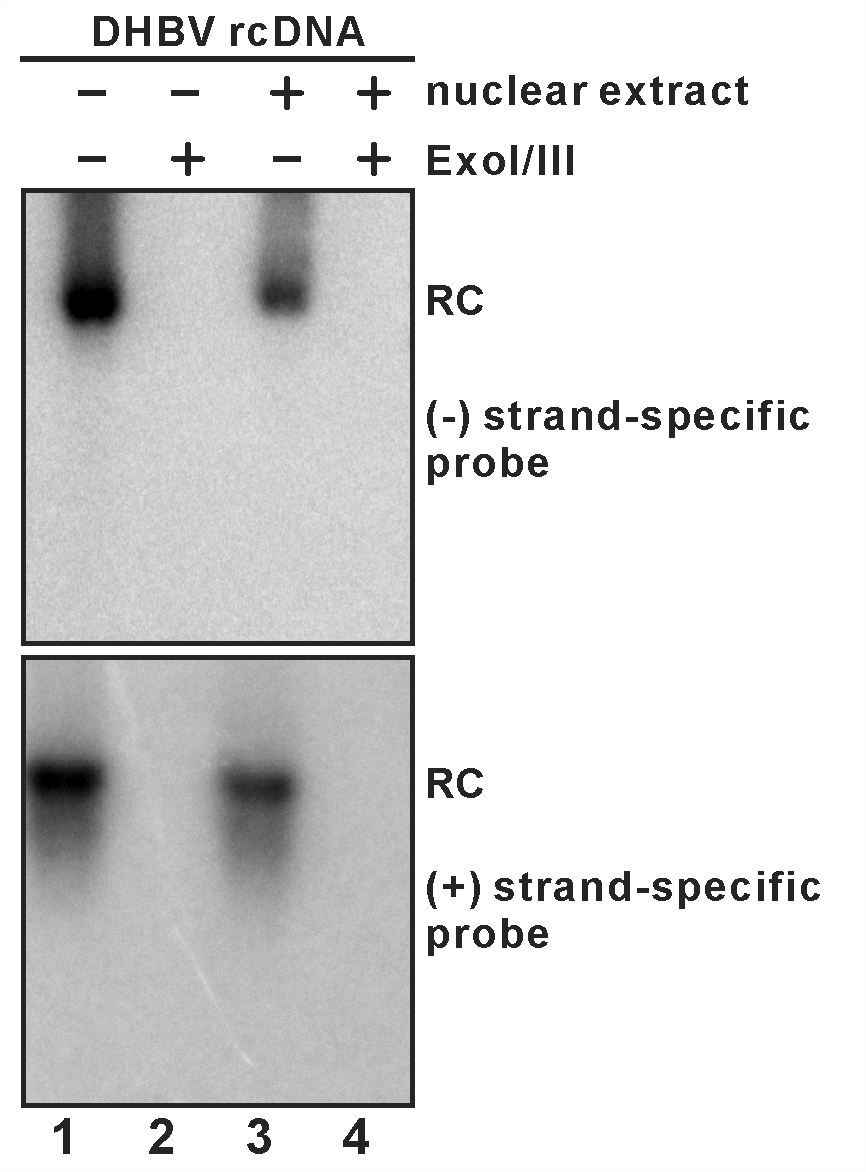

Supplement: S3 Fig — DHBV rcDNA (5 ng) were left untreated (lane 1) or treated with ExoI/III (lane 2) as described in Materials and Methods. In addition, 5 ng of DHBV rcDNA were subjected to the in vitro cccDNA formation reaction and the recovered DNA were left undigested (lane 3) or digested by ExoI/III (lane 4). The digestion products were analyzed by Southern blot hybridization with DHBV minus- or plus-strand specific riboprobe. (TIF) [file ppat.1006784.s003.tif]

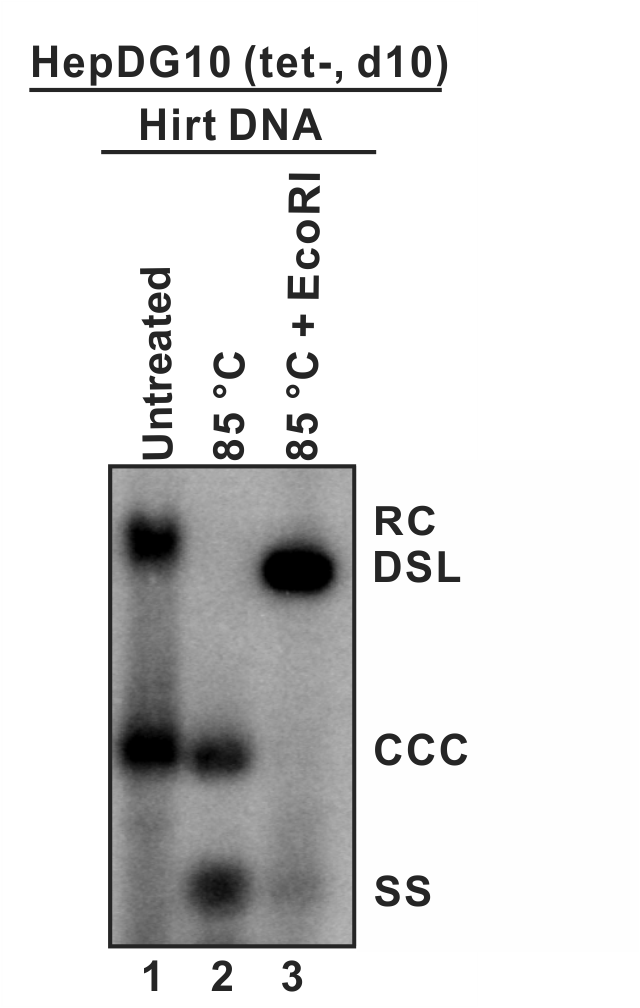

Supplement: S4 Fig — cccDNA produced in HepDG10 cells at day 10 post induction was extracted by Hirt extraction and subjected to Southern blot hybridization. To further validate the authenticity of DHBV cccDNA, Hirt DNA samples were heated to 85°C for 5 min before gel loading, a condition that denatures rcDNA into ssDNA, while the cccDNA stays undenatured and its electrophoretic mobility remains unchanged (lanes 1–2). The heat denatured DNA samples were further digested with EcoRI, by which the cccDNA was linearized to double-stranded DNA (lane 3). (TIF) [file ppat.1006784.s004.tif]

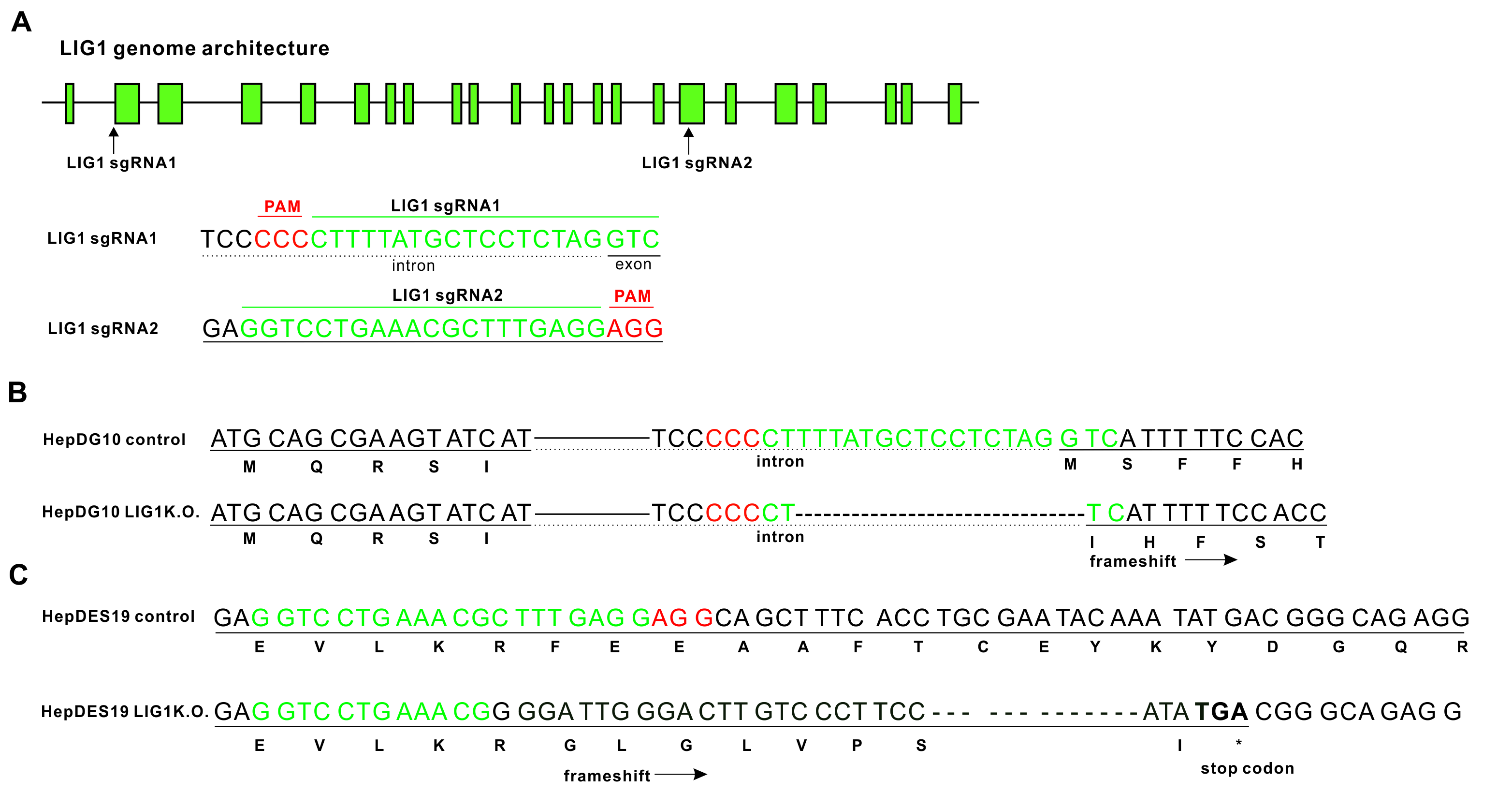

Supplement: S5 Fig — (A) Schematic illustration of LIG1 gene locus. The green boxes indicate exons and the solid lines indicate introns. Two designed sgRNA are shown in nucleotide sequence (green) with adjacent PAM sequence shown in red, and their corresponding targeting sites in LIG1 gene are marked. sgRNA1 and sgRNA2 were used to CRISPR out LIG1 in HepDG10 and HepDES19 cells, respectively. The procedure for making LIG1 knock-out cells by CRISPR/Cas9 system is described in Materials and Methods. (B) Indel sequencing assay of HepDG10 cells with LIG1 knock-out. DNA fragment spanning the sgRNA1 targeting region of LIG1 gene loci was PCR amplified from the control and LIG1 knock-out HepDG10 cells and sequenced. The nucleotide sequences of wildtype and CRISPR-mutated LIG1 gene fragment are aligned. The nucleotides within exons are underlined. The omitted sequence of the intron was indicated with a solid line. The deletion mutations of CRISPRed LIG1 gene is shown as dashed line. The mutation-mediated disruption and frame shift of LIG1 ORF are depicted with amino acid codes. (C) Indel sequencing assay of LIG1 deficient HepDES19 cells. Alignment of the sgRNA2 targeting region in LIG1 gene loci from the control and LIG1 knock-out HepDES19 cells shows indels, frameshift, and premature termination of LIG1 ORF in the knock-out cells. (TIF) [file ppat.1006784.s005.tif]

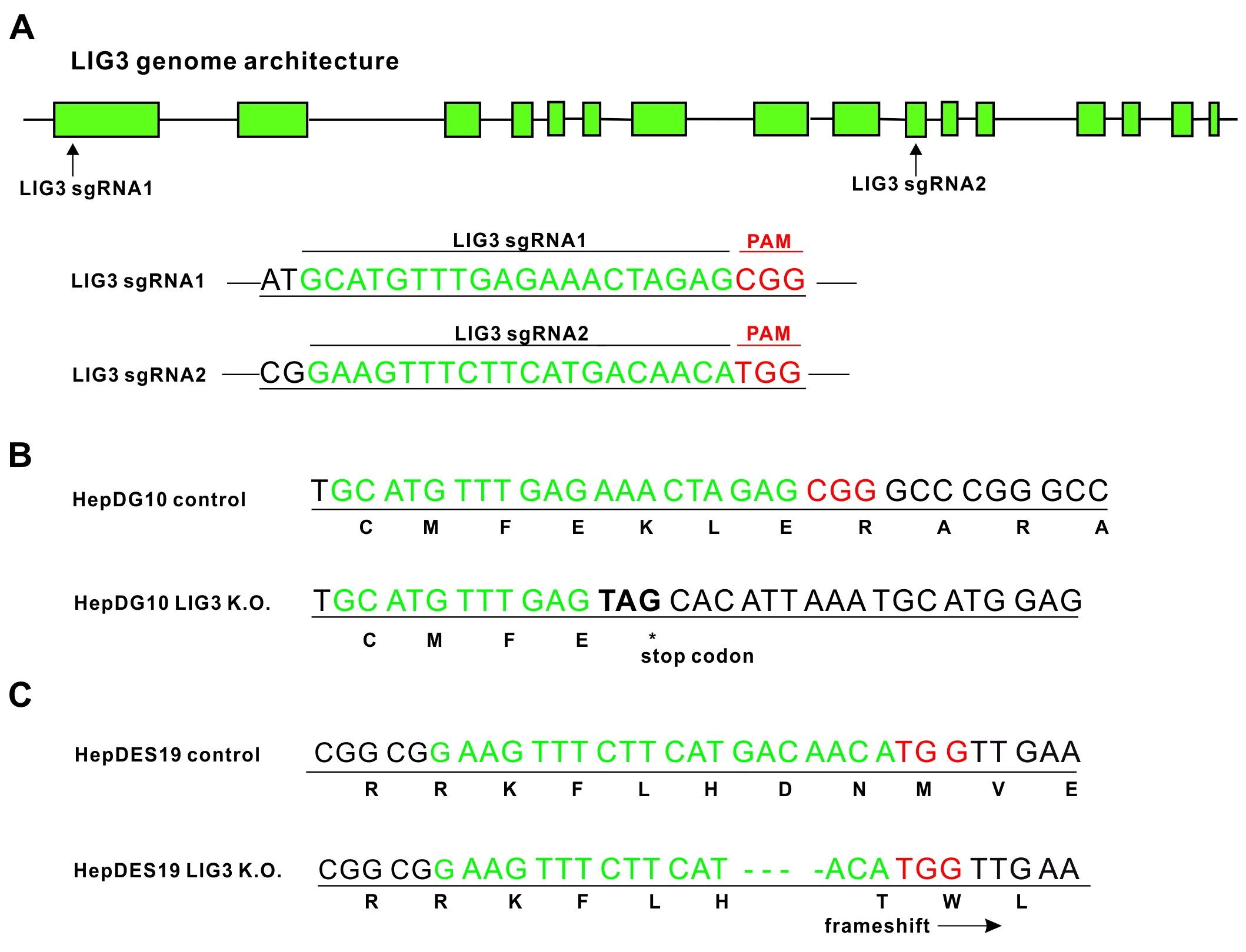

Supplement: S6 Fig — (A) Schematic illustration of LIG3 gene locus, exons are shown in green rectangles. The designed sgRNAs with PAM motif are shown in nucleotide sequence. (B) Indel sequencing analysis of HepDG10 LIG3 knock-out cells. Compared to the wildtype LIG3 sequence in HepDG10 control knock-out cells, a stop codon was introduced into the exon1 of LIG3 gene loci in HepDG10 LIG3 knock-out cells by sgRNA1-guided CRISPR editing. (C) Indel sequencing analysis of HepDES19 LIG3 knock-out cells. The sequencing result demonstrated a frameshift mutation of LIG3 ORF in HepDES19 LIG3 knock-out cells. (TIF) [file ppat.1006784.s006.tif]

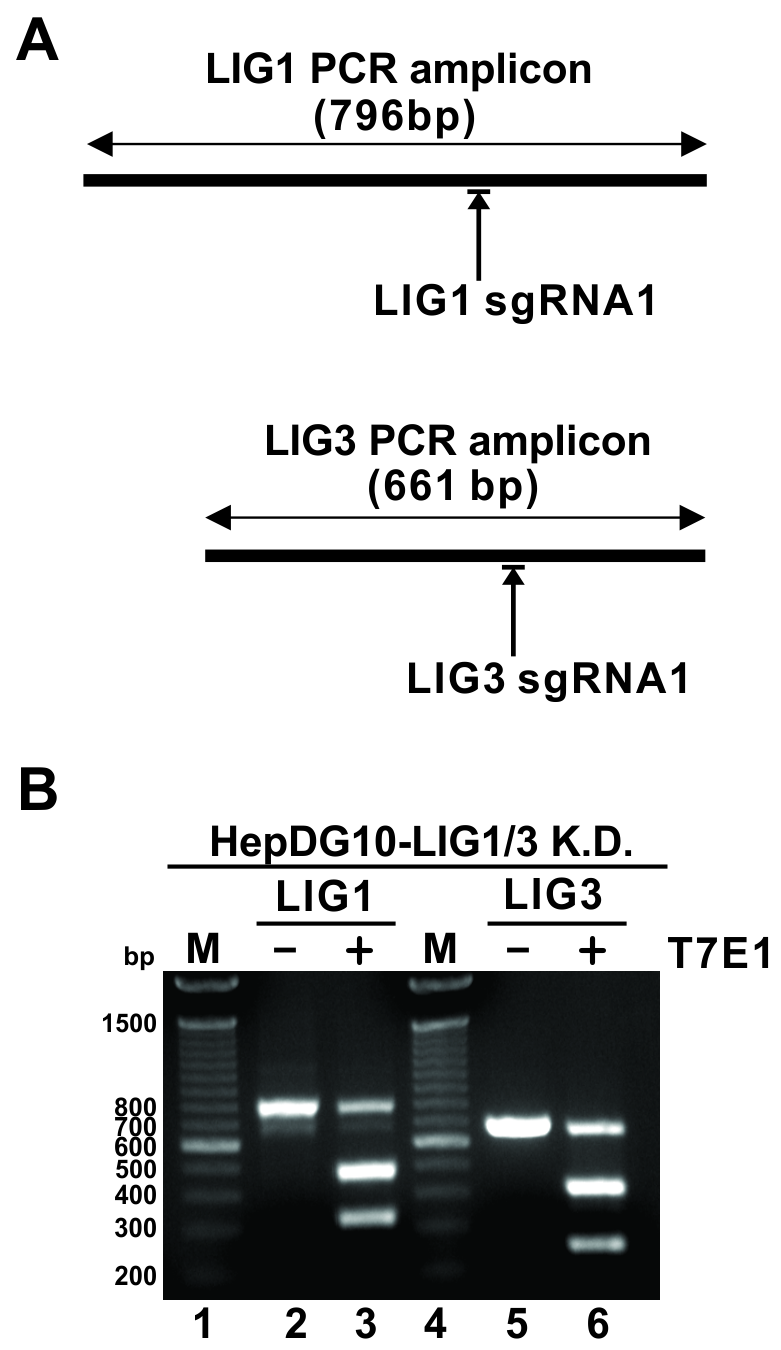

Supplement: S7 Fig — (A) Schematic illustration of the PCR amplicons of LIG1 and LIG3 DNA fragments containing the corresponding sgRNA targeting region. The sequences of sgRNA and PCR primers are listed in S2 and S3 Tables, respectively. (B) The DNA fragments were PCR amplified from genomic DNA of HepDG10-LIG1/3 K.D. cells transduced by CRISPR/Cas9-LIG1/3 sgRNA, the indel mutations were detected by T7E1 digestion. (TIF) [file ppat.1006784.s007.tif]

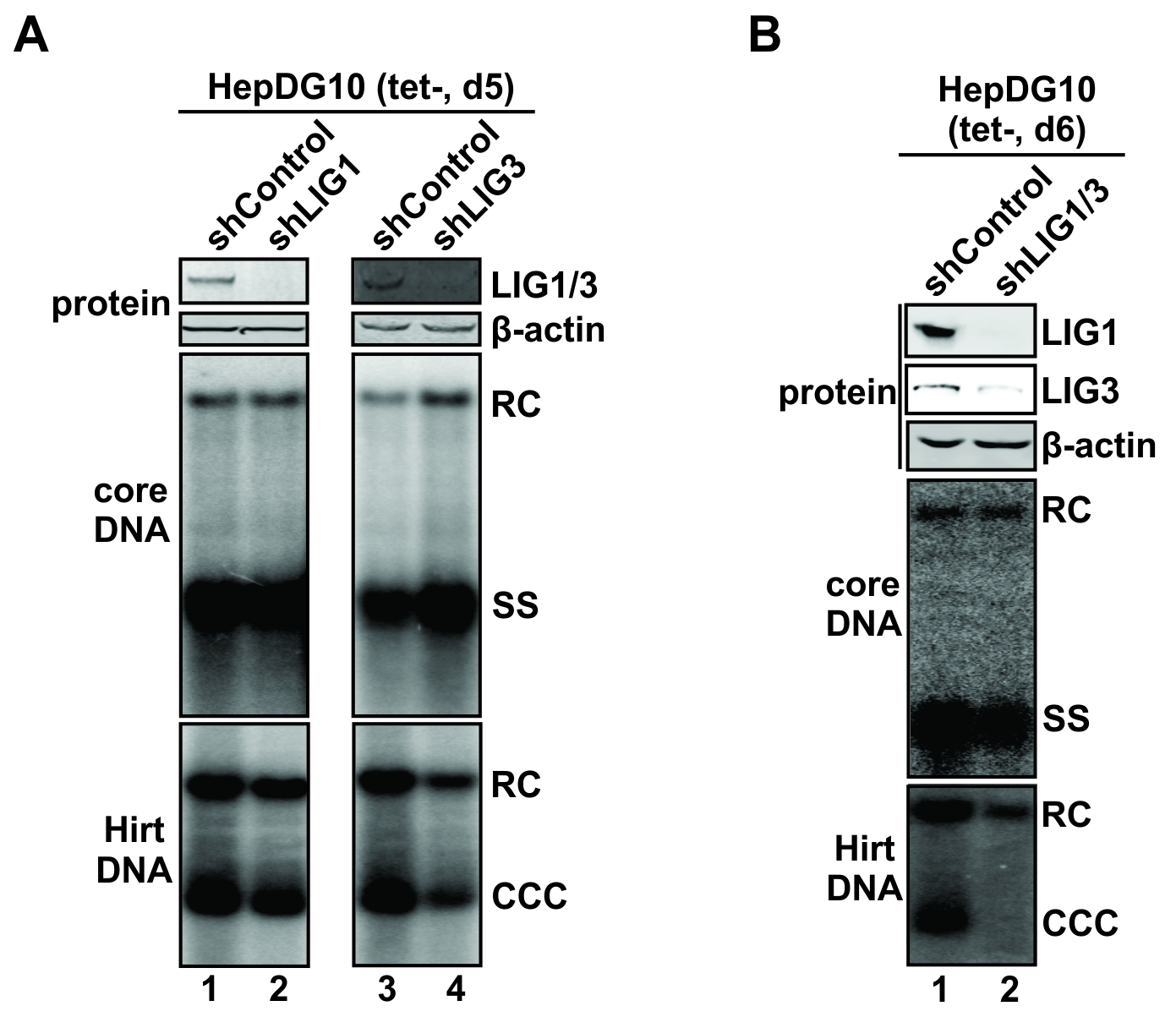

Supplement: S8 Fig — HepDG10 control and ligase single knock-down cells (A) and double knock-down cells (B) were cultured in tet-free medium for the indicated duration. The levels of LIG1 and LIG3 in each cell lines were determined by Western blot with β-actin serving as loading control. DHBV core DNA and cccDNA were analyzed by Southern blot. The loading amount of Hirt DNA was normalized based on the relative levels of RC DNA (% of control) on core DNA Southern blot. (TIF) [file ppat.1006784.s008.tif]

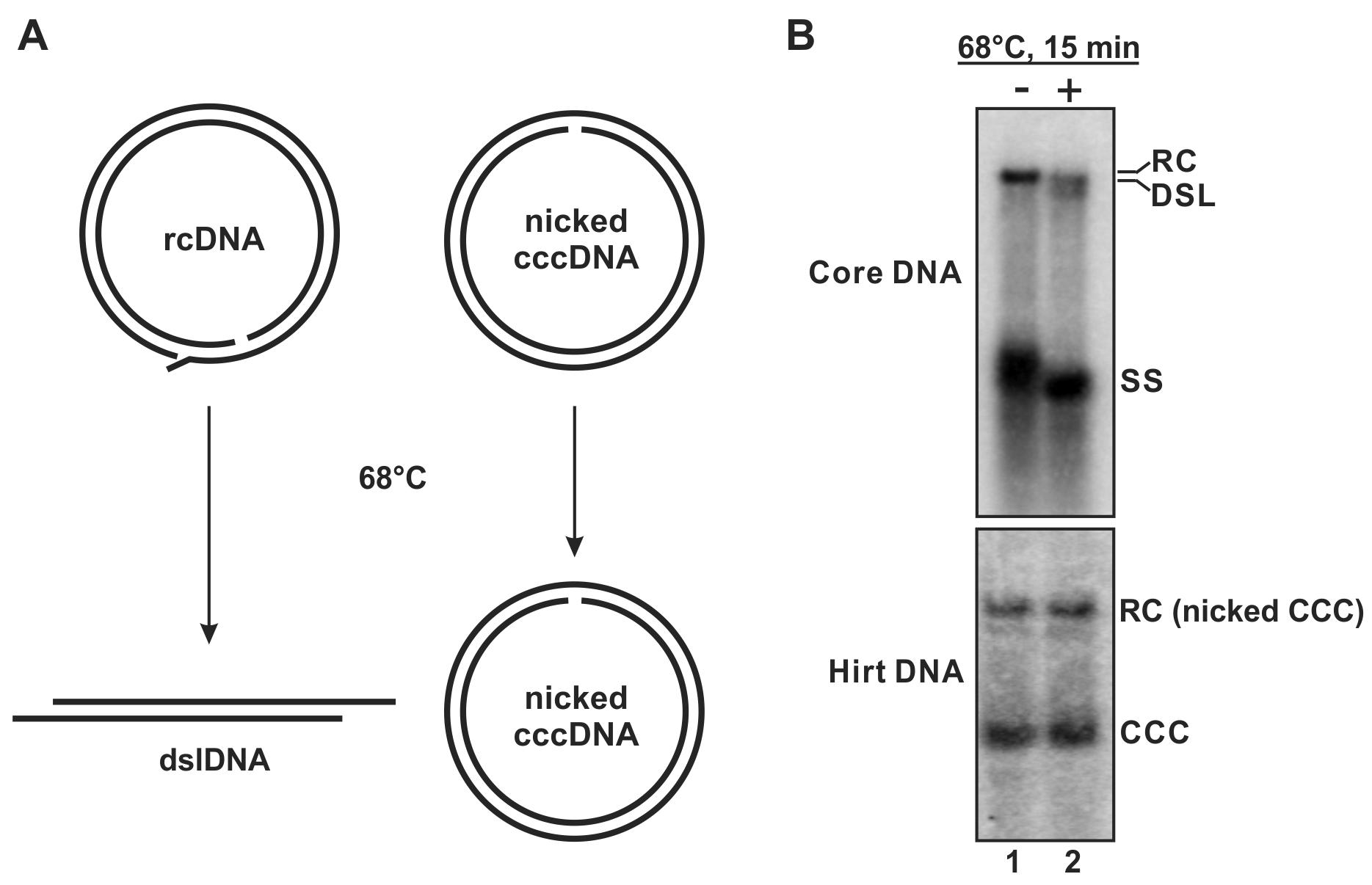

Supplement: S9 Fig — (A) Schematic illustration of the conformational change of true rcDNA and nicked cccDNA in response to mild heat treatment. When heating up to 68°C, the cohesive end of rcDNA between two single strand gaps is melted and the rcDNA is converted into dslDNA, while the nicked cccDNA remains resistant to heat. (B) The cytoplasmic DHBV core DNA and whole cell Hirt DNA extracted from HepDG10 cells (tet-, d6) were left untreated or heated under 68°C for 15 min, followed by Southern blot analysis. (TIF) [file ppat.1006784.s009.tif]

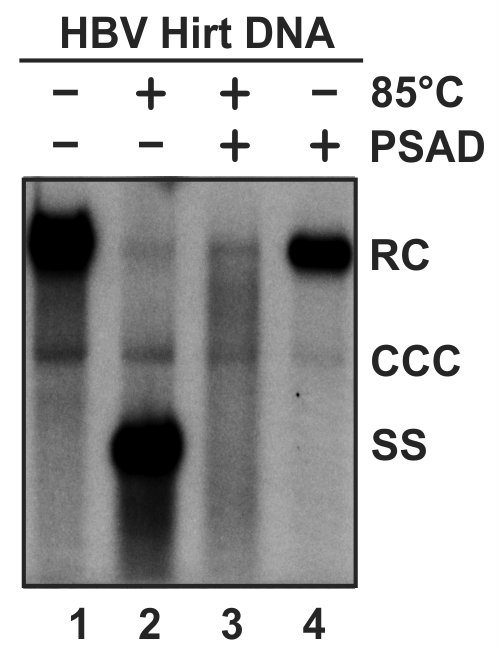

Supplement: S10 Fig — Hirt DNA extracted from HepDES19 cells cultured in tet-free medium was directly subjected to electrophoresis (lane 1), or pretreated by heat denaturation at 85°C for 5 min before gel loading (lane 2), or digested by PSAD with or without heat denaturation prior to electrophoresis (lanes 3 and 4). The HBV DNA signals were detected by Southern blot. (TIF) [file ppat.1006784.s010.tif]

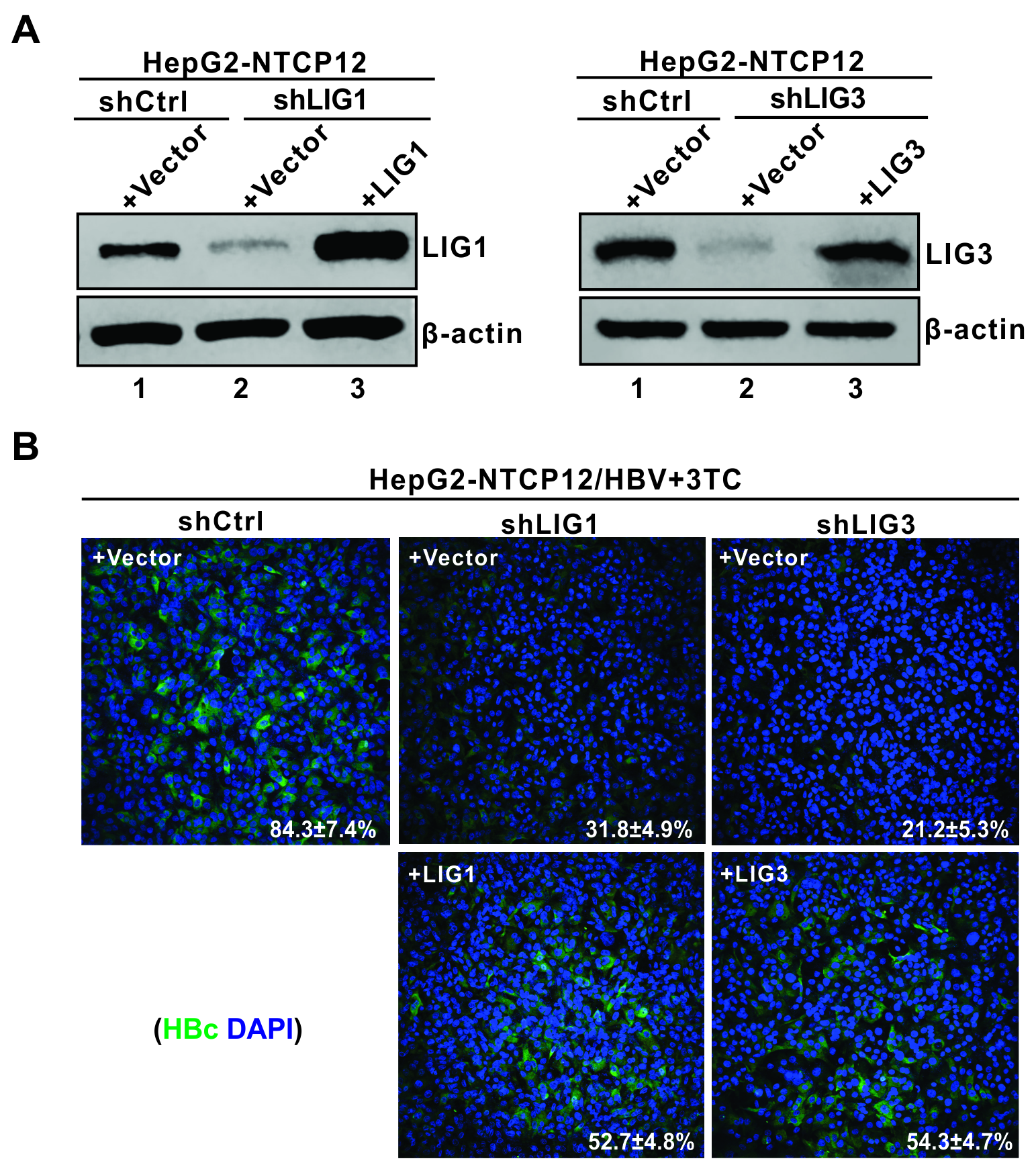

Supplement: S11 Fig — HepG2-NTCP12 shControl (shCtrl), shLIG1, and shLIG3 cells were transfected with empty vector, or plasmid expressing wild-type LIG1 or LIG3, as indicated. Followed by HBV infection in the presence of 10 μM 3TC for 6 days. (A) The expression levels of LIG1 and LIG3 were determined by Western blot. β-actin served as loading control. (B) The intracellular core protein (HBc) expression was detected by immunofluorescence microscopy. Each image shown here represents five different microscopic fields, the percentage of HBc positive cells was indicated (Mean ± SD). (TIF) [file ppat.1006784.s011.tif]

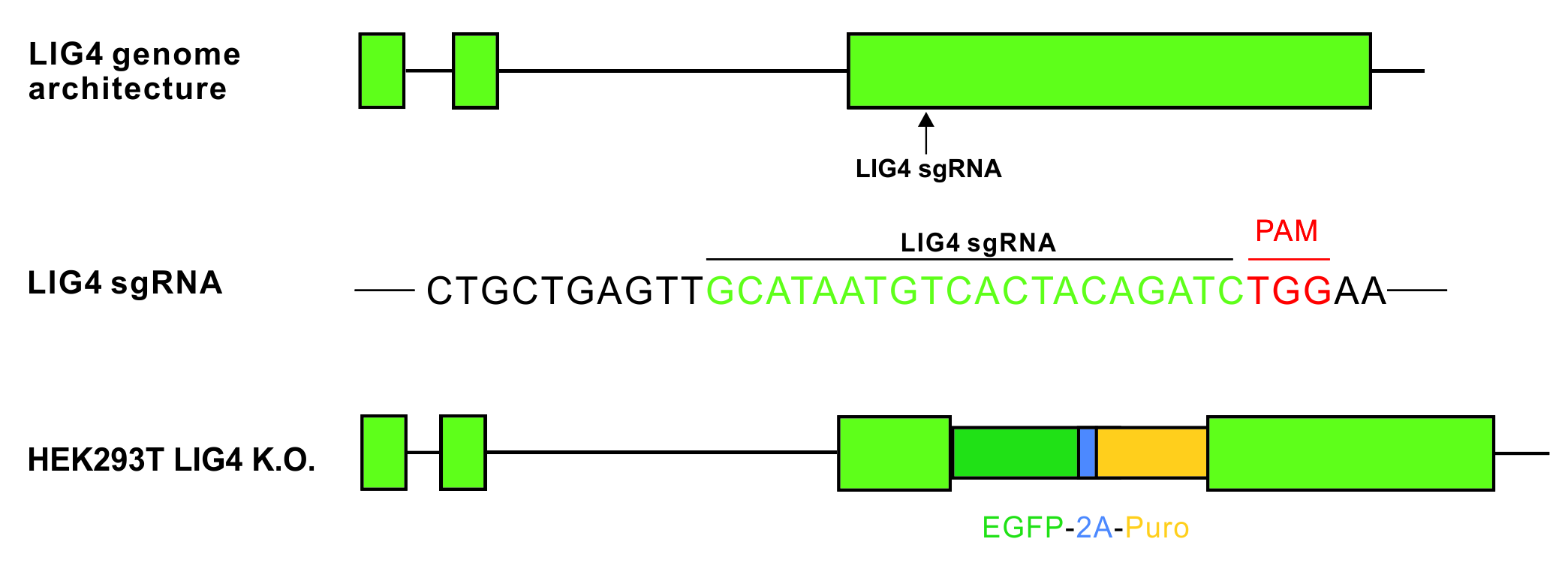

Supplement: S12 Fig — The exons of LIG4 gene locus are shown in green boxes. The sequence of LIG4 sgRNA is shown and its targeting site in exon 3 of LIG4 is marked. The MMEJ-based CRISPR PITCh system will knock in an EGFP-2A-Puro cassette into the sgRNA targeting site of LIG4 ORF to disrupt gene expression. Refer to Materials and Methods for detailed experimental procedures. (TIF) [file ppat.1006784.s012.tif]
