# Supplementary material for: The role of host DNA ligases in hepadnavirus covalently closed circular DNA formation
Source: PLoS Pathog. 2017 Dec 29;13(12):e1006784. doi: 10.1371/journal.ppat.1006784 (PMC5747486; doi:10.1371/journal.ppat.1006784)
Supplement: S1 Table — (PDF) [file ppat.1006784.s013.pdf]

S1 Table. shRNA sequence for human LIG1 and LIG3.

Gene	Sequence (5’→3’ orientation)
LIG1	CCGGCGGTTTATTCGAGTCCGTGAACTCGAGTTCACGGACTCGAATAAACCGTTTTTG
LIG3	CCGGGCCCACTTTAAGGACTACATTCTCGAGAATGTAGTCCTTAAAGTGGGCTTTTTG
