# Supplementary material for: The role of host DNA ligases in hepadnavirus covalently closed circular DNA formation
Source: PLoS Pathog. 2017 Dec 29;13(12):e1006784. doi: 10.1371/journal.ppat.1006784 (PMC5747486; doi:10.1371/journal.ppat.1006784)
Supplement: S2 Table — (PDF) [file ppat.1006784.s014.pdf]

S2 Table. Oligos for LIG1/3 CRISPR sgRNA.

Name	Sequence (5’→3’ orientation)
**HepDG10 cells**	
LIG1 sgRNA1 forward (F)	caccgGACCTAGAGGAGCATAAAAG
LIG1 sgRNA1 reverse (R)	aaacCTTTTATGCTCCTCTAGGTCc
LIG3 sgRNA1 forward (F)	caccgGCATGTTTGAGAAACTAGAG
LIG3 sgRNA1 reverse (R)	aaacCTCTAGTTTCTCAAACATGCc
**HepDES19 cells**	
LIG1 sgRNA2 forward (F)	caccgGGTCCTGAAACGCTTTGAGG
LIG1 sgRNA2 reverse (R)	aaacCCTCAAAGCGTTTCAGGACCc
LIG3 sgRNA2 forward (F)	caccgGAAGTTTCTTCATGACAACA
LIG3 sgRNA2 reverse (R)	aaacTGTTGTCATGAAGAAACTTCc
