# Supplementary material for: The role of host DNA ligases in hepadnavirus covalently closed circular DNA formation
Source: PLoS Pathog. 2017 Dec 29;13(12):e1006784. doi: 10.1371/journal.ppat.1006784 (PMC5747486; doi:10.1371/journal.ppat.1006784)
Supplement: S3 Table — (PDF) [file ppat.1006784.s015.pdf]

S3 Table. Oligos for LIG1/3 sgRNA indel sequencing and T7E1 assay.

Name	Sequence (5'→3' orientation)
**HepDG10 cells**	
LIG1 sgRNA1 indel sequencing forward (F)	ACATTGGGAGGCCAAGCAGATCACA
LIG1 sgRNA1 indel sequencing reverse (R)	GGGCCCCAAGACATTTTGCATTAG
LIG3 sgRNA1 indel sequencing forward (F)	TGTCTTTGGCTTTCAAGATCTTCTTTCC
LIG3 sgRNA1 indel sequencing reverse (R)	ACATTAAACACTGGCCTAGGCAACAGA
LIG1 sgRNA1 T7E1 forward (F)	TGCCTCCTTTGTCTGAGTCTG
LIG1 sgRNA1 T7E1 reverse (R)	CTAGAACGAGGCCAAGACCTG
LIG3 sgRNA1 T7E1 forward (F)	TCTTTGGCTTTCAAGATCTTCTTTC
LIG3 sgRNA1 T7E1 reverse (R)	GTGAGACCCCATCTCTAAAAGAGC
**HepDES19 cells**	
LIG1 sgRNA2 indel sequencing forward (F)	CCTGTTCTAATTGTGGGAGGAGG
LIG1 sgRNA2 indel sequencing reverse (R)	TGCTGAAGCCCAGAGAGATGA
LIG3 sgRNA2 indel sequencing forward (F)	CCAGTTCTCCCTTGGCTCGCTT
LIG3 sgRNA2 indel sequencing reverse (R)	GGGACAAGAGAGATGCTGAAGTTGGTGT
