# Supplementary material for: The role of host DNA ligases in hepadnavirus covalently closed circular DNA formation
Source: PLoS Pathog. 2017 Dec 29;13(12):e1006784. doi: 10.1371/journal.ppat.1006784 (PMC5747486; doi:10.1371/journal.ppat.1006784)
Supplement: S4 Table — (PDF) [file ppat.1006784.s016.pdf]

S4 Table. Oligos for LIG4 sgRNA and knock-in.

Name	Sequence (5'→3' orientation)
LIG4 sgRNA2 MMEJ sense	cttcGCATAATGTCACTACAGATC
LIG4 sgRNA2 MMEJ antisense	aaacGATCTGTAGTGACATTATGC
5'FWD_LIG4-2-MMEJ	CCGCGTTACATAGCATCGTACGCGTACGTGTTTGGGTTGCATAATGTCACTACAGCCGGATCCATGGTGAGCAAGGG
3'REV_LIG4-2-MMEJ	ACGCGTACGTGTTTGGCCTACAGACTTTTTCCAGATTCAGGCACCGGGCTTGCG
5'REV_generic	TGCTATGTAACGCGGAACTCCATATATGGG
3'FWD_generic	CAAACACGTACGCGTACGATGCTCTAGAATG
